# Supplementary material for: Associations between components of household expenditures and the rate of change in the number of new confirmed cases of COVID-19 in Japan: Time-series analysis
Source: PLoS One. 2022 Apr 14;17(4):e0266963. doi: 10.1371/journal.pone.0266963 (PMC9009719; doi:10.1371/journal.pone.0266963)
Supplement: S1 Table — (PDF) [file pone.0266963.s001.pdf]

S1 Table. Japanese names of household expenditure variables in the Family Income and Expenditure Survey and the Consumer Price Index.

English name in the main text of the paper	Name in the Family Income and Expenditure Survey	Name in the Consumer Price Index
Meals at bars and restaurants	Shokuji-dai	–
Soft drinks, confectioneries, and fruits at bars and restaurants	Kissa-dai	–
Alcoholic drinks at bars and restaurants	Inshu-dai	–
Food consumption at bars and restaurants in general	Ippan-gaishoku	
Non-packaged lodging	Shukuhaku-ryo	–
Domestic travel packages	Kokunai-pakku-ryoko-hi	–
Lodging	–	Shukuhaku-ryo
Admissions, viewing, and game fees	Nyujyo · kanran · gemu-dai	
Clothing and footwear	Hifuku oyobi hakimono	
Food	Shokuryo	
Housing	Jyukyo	
Fuel, light, and water charges	Konetsu · Suido	
Furniture and household utensils	Kaji · Kaji-yohin	
Medical care	Hoken-iryo	
Transportation and communication	Kotsu · Tsushin	
Education	Kyoiku	
Culture and recreation	Kyoyo-goraku	
The other household consumption expenditures	Sonota no shohi shishutsu	

Notes: "–" indicates there is no corresponding category in the dataset. "The other household consumption expenditures" in the table indicates one of the large categories of household expenditures, rather than one of the explanatory variables in the regression model.
