# Supplementary material for: Associations between components of household expenditures and the rate of change in the number of new confirmed cases of COVID-19 in Japan: Time-series analysis
Source: PLoS One. 2022 Apr 14;17(4):e0266963. doi: 10.1371/journal.pone.0266963 (PMC9009719; doi:10.1371/journal.pone.0266963)
Supplement: S2 Table — (PDF) [file pone.0266963.s002.pdf]

S2 Table. Posterior mean and the credible interval of each parameter in the regression model.

Notation of explanatory variables:

$X_{1,t}$	Meals at bars and restaurants
$X_{2,t}$	Soft drinks, confectioneries, and fruits at bars and restaurants
$X_{3,t}$	Alcoholic drinks at bars and restaurants
$X_{4,t}$	Non-packaged lodging
$X_{5,t}$	Domestic travel packages
$X_{6,t}$	Admissions, viewing, and game fees
$X_{7,t}$	Clothing and footwear
$X_{8,t}$	The other household consumption expenditures
$X_{9,t}$	transit_stations for Japan in the COVID-19 Community Mobility Reports

$D_{NY,t}$	Time dummy for December 29, 2020 - January 3, 2021
$D_{AH,t}$	Nationwide dummy for absolute humidity
$D_{SoE,1,t}$	Time dummy for the period before the first state of emergency (- April 6, 2020)
$D_{SoE,2,t}$	Time dummy for the first state of emergency (April 7 - May 25, 2020)
$D_{SoE,3,t}$	Time dummy for the second state of emergency (January 7 - March 21, 2021)

Parameter estimates:

	Posterior mean	2.5%	97.5%		Posterior mean	2.5%	97.5%
α_0	-0.814	-1.636	-0.069	ψ_{11}	0.051	-0.093	0.289
α_1	0.592	0.026	1.684	ψ_{12}	0.817	-1.491	4.526
α_2	-0.117	-0.425	-0.003	ψ_{13}	-0.213	-2.218	2.099
β_1	-2.196	-4.834	-0.068	ψ_{14}	0.316	-0.453	1.504
β_2	-1.203	-4.620	2.081	ψ_{15}	0.131	-0.285	0.787
β_3	-1.040	-7.083	5.041	ψ_{16}	0.570	-0.257	2.183
γ_1	0.060	0.006	0.167	ψ_{17}	0.251	-0.098	0.703
γ_2	1.038	0.126	2.914	ψ_{18}	0.017	-0.007	0.055
γ_3	1.339	0.172	3.120	ψ_{19}	0.000	-0.018	0.020
γ_4	0.356	0.047	0.912	ψ_{21}	0.177	-0.064	0.674
γ_5	0.221	0.029	0.608	ψ_{22}	5.302	-0.642	17.590
γ_6	0.266	0.029	0.768	ψ_{23}	1.022	-1.910	5.766
γ_7	0.102	0.012	0.261	ψ_{24}	7.789	0.300	18.478
γ_8	0.006	0.001	0.017	ψ_{25}	3.277	-0.037	10.964
γ_9	0.019	0.004	0.042	ψ_{26}	3.168	0.029	8.753
θ_1	-0.018	-0.068	-0.000	ψ_{27}	0.141	-0.148	0.651
θ_2	-0.394	-1.432	-0.009	ψ_{28}	0.026	-0.005	0.076
θ_3	-0.231	-0.844	-0.006	ψ_{29}	0.025	-0.010	0.071
θ_4	-0.135	-0.481	-0.003	ψ_{31}	0.338	-0.052	1.221
θ_5	-0.100	-0.361	-0.002	ψ_{32}	15.980	0.012	45.955
θ_6	-0.120	-0.468	-0.003	ψ_{33}	12.249	-0.596	34.309
θ_7	-0.022	-0.078	-0.001	ψ_{34}	1.854	-0.250	6.561
θ_8	-0.002	-0.005	-0.000	ψ_{35}	3.748	0.054	10.923
θ_9	-0.012	-0.029	-0.001	ψ_{36}	4.277	0.012	13.293
ρ	0.756	0.638	0.875	ψ_{37}	0.307	-0.115	1.102
σ	0.312	0.287	0.341	ψ_{38}	0.029	-0.006	0.100
				ψ_{39}	0.162	0.010	0.367

Notes: "2.5%" and "97.5%" indicate the percentiles of MCMC samples.
