# Supplementary material for: Associations between components of household expenditures and the rate of change in the number of new confirmed cases of COVID-19 in Japan: Time-series analysis
Source: PLoS One. 2022 Apr 14;17(4):e0266963. doi: 10.1371/journal.pone.0266963 (PMC9009719; doi:10.1371/journal.pone.0266963)
Supplement: S1 Appendix — (PDF) [file pone.0266963.s003.pdf]

S1 Appendix. How to construct the consumer price index for each explanatory variable on household expenditures.

The Consumer Price Index (CPI) published by the Statistics Bureau in the Ministry of Internal Affairs and Communications, Japan, contains a monthly price index for each category of consumer goods and services. Because consumer price indices are not available at daily frequency, the monthly average of each consumer price index is used for each date in the same month.

Because there are no separate consumer price indices corresponding to meals at bars and restaurants, soft drinks, confectioneries, and fruits at bars and restaurants, and alcoholic drinks at bars and restaurants, the consumer price index for food consumption at bars and restaurants in general is used to divide nominal household expenditures per household for the three types of food consumption at bars and restaurants among the explanatory variables.

Because there is no consumer price index corresponding to domestic travel packages, the consumer price index for lodging is used to divide nominal household expenditures per household for domestic travel packages to compute their real value on each date. Consumer price indices for transportation are not used here, because they include not only traveling, but also commuting.

To compute the real value of household expenditures per household for non-packaged lodging, the effect of the Go-To-Travel campaign must be removed from the consumer price index for lodging. Therefore, when the consumer price index for lodging is used to construct a consumer price index for non-packaged lodging, it is interpolated between July 2020 and January 2021 to calculate the values of the consumer price index for non-packaged lodging from August to December 2020.

There exist corresponding consumer price indices for admissions, viewing, and game fees, and clothing and footwear.

The consumer price index for "the other household consumption expenditures" among the explanatory variables is constructed as follows. The consumer price index for each category of goods and services has a fixed weight, which is computed from the average share of household expenditures per household for the corresponding category of goods and services. This weight is available from the dataset. Each index is normalized to 100 for the base month. Then, the consumer price index for all goods and services is computed as the weighted average of the consumer price indices of all categories of goods and services:

$$\text{Consumer price index for all goods and services} = \frac{\sum_{i=1}^{I} p_{i,t} w_i}{\sum_{i=1}^{I} w_i}$$

where p_i is the consumer price index for the i-th category of goods and services, w_i is its weight, and I is the total number of indices. For "the other household consumption expenditures" among the explanatory variables, the consumer price index is computed as the weighted average of consumer price indices for the other items than food consumption at bars and restaurants in general; lodging; admissions, viewing, and game fees; and clothing

and footwear:

$$\text{Consumer price index for “the other household consumption expenditures”} = \frac{\sum_{i \in S} p_{i,t} w_i}{\sum_{i \in S} w_i}$$

where S is the set of indices for the other items than food consumption at bars and restaurants in general; lodging; admissions, viewing, and game fees; and clothing and footwear.
