# Supplementary material for: Associations between components of household expenditures and the rate of change in the number of new confirmed cases of COVID-19 in Japan: Time-series analysis
Source: PLoS One. 2022 Apr 14;17(4):e0266963. doi: 10.1371/journal.pone.0266963 (PMC9009719; doi:10.1371/journal.pone.0266963)
Supplement: S2 Appendix — (PDF) [file pone.0266963.s004.pdf]

S2 Appendix. The formula to compute absolute humidity.

$$AH = \frac{6.112 \cdot \exp\left(\frac{17.67 \cdot t}{t+243.5}\right) \cdot h \cdot 2.1674}{273.15 + t}$$

where AH denotes the absolute humidity, t is the Celsius temperature, and h is the relative humidity. See Nottmeyer and Sera (2021) [38] for more details.
