# Supplementary material for: Associations between components of household expenditures and the rate of change in the number of new confirmed cases of COVID-19 in Japan: Time-series analysis
Source: PLoS One. 2022 Apr 14;17(4):e0266963. doi: 10.1371/journal.pone.0266963 (PMC9009719; doi:10.1371/journal.pone.0266963)
Supplement: S3 Appendix — (PDF) [file pone.0266963.s005.pdf]

S3 Appendix. How to fulfill missing values in Celsius temperature and relative humidity data published by the Japan Meteorological Agency.

The Japan Meteorological Agency (JMA) does not publish relative humidity data for the capitals of Saitama and Shiga prefecture. Relative humidity data for the capitals of Tokyo and Kyoto prefecture are substituted for the missing values for Saitama and Shiga prefecture, respectively, because of the geographical proximity of capitals between each pair of prefectures.

In addition, relative humidity data are missing for the capitals of Hyogo, Kumamoto, and Kanagawa prefecture on May 6, 2020, December 10, 2020, and June 20, 2021, respectively. The value of relative humidity for each pair of a location and a date is fulfilled by the average value of relative humidity between the previous date and the next date at the same location.
