# Supplementary material for: Associations between components of household expenditures and the rate of change in the number of new confirmed cases of COVID-19 in Japan: Time-series analysis
Source: PLoS One. 2022 Apr 14;17(4):e0266963. doi: 10.1371/journal.pone.0266963 (PMC9009719; doi:10.1371/journal.pone.0266963)
Supplement: S4 Appendix — (PDF) [file pone.0266963.s006.pdf]

S4 Appendix Sensitivity analysis with alternative weather variables for explanatory variables.

1. Construction of alternative weather variables for explanatory variables

To construct the nationwide dummy for outside temperature, a dummy for Celsius outside temperature being no less than $18°C$ is computed for the capital of each prefecture in Japan on each date, using Celsius temperature published by the Japan Meteorological Agency (`https://www.data.jma.go.jp/gmd/risk/obsdl/index.php`). These dummies are weighted by the population of each prefecture in 2019, and then summed across prefectures to compute the population-weighted nationwide average of the prefectural dummies on each date. The population of each prefecture in 2019 is published by the Ministry of Internal Affairs and Communications, the Government of Japan (`https://www.stat.go.jp/english/data/jinsui/2019np/index.html`).

To construct the nationwide averages of absolute humidity and outside temperature, the absolute humidity and outside temperature at the capital of each prefecture are weighted by the population of each prefecture in 2019. Each variable is summed across prefectures to compute the population-weighted nationwide average on each date. See S3 Appendix for the formula to compute absolute humidity.

2. Estimation of the regression model with alternative weather variables for explanatory variables

The regression model is estimated with each of the four variables substituting $D_{AH,t}$ in Eq (7): the nationwide dummy for outside temperature; the nationwide average of absolute humidity; the nationwide average of outside temperature; and no weather variable. Tables S4.1-S4.4 show parameter estimates for each regression. Figures S4.1, S4.2, S4.3 show the time series of the nationwide dummy for outside temperature, the nationwide average of absolute humidity, and the nationwide average of outside temperature, respectively. For each set of explanatory variables, Figures S4.4-S4.7 show the fitted values and out-of-sample forecasts of the regression; Figures S4.8-S4.11 show the decomposition of out-of-sample forecasts into contributions from explanatory variables; and Figures S4.12-S4.15 show the decomposition of fitted values into contributions from explanatory variables.

Table S4.1: Estimates of parameters in the regression model with the nationwide dummy for outside temperature substituting $D_{AH,t}$ in Eq (7)

	Posterior mean	2.5%	97.5%		Posterior mean	2.5%	97.5%
α_0	-0.845	-1.676	-0.074	ψ_{11}	0.187	-0.066	0.726
α_1	0.522	0.018	1.507	ψ_{12}	5.705	-0.758	19.578
α_2	-0.133	-0.550	-0.003	ψ_{13}	1.064	-1.867	5.812
β_0	-2.374	-5.036	-0.174	ψ_{14}	6.948	0.167	17.109
β_1	-1.566	-5.261	1.743	ψ_{15}	3.387	-0.029	10.860
β_2	-1.182	-7.198	4.829	ψ_{16}	3.083	-0.032	8.730
γ_1	0.063	0.007	0.175	ψ_{17}	0.197	-0.125	0.773
γ_2	1.078	0.128	2.974	ψ_{18}	0.026	-0.005	0.077
γ_3	1.294	0.189	3.041	ψ_{19}	0.022	-0.012	0.069
γ_4	0.369	0.047	0.926	ψ_{21}	0.354	-0.046	1.246
γ_5	0.231	0.031	0.644	ψ_{22}	15.971	-0.060	47.176
γ_6	0.275	0.030	0.808	ψ_{23}	12.380	-0.506	35.216
γ_7	0.095	0.012	0.245	ψ_{24}	1.780	-0.274	6.105
γ_8	0.006	0.001	0.016	ψ_{25}	3.712	0.053	10.960
γ_9	0.015	0.002	0.037	ψ_{26}	4.195	-0.005	13.030
θ_1	-0.019	-0.075	-0.000	ψ_{27}	0.315	-0.107	1.110
θ_2	-0.395	-1.529	-0.009	ψ_{28}	0.030	-0.005	0.099
θ_3	-0.266	-1.005	-0.008	ψ_{29}	0.163	0.011	0.372
θ_4	-0.141	-0.516	-0.003	ψ_{01}	0.055	-0.098	0.315
θ_5	-0.098	-0.363	-0.003	ψ_{02}	0.942	-1.578	5.166
θ_6	-0.117	-0.455	-0.003	ψ_{03}	0.026	-2.120	2.636
θ_7	-0.023	-0.086	-0.001	ψ_{04}	0.316	-0.469	1.532
θ_8	-0.002	-0.006	-0.000	ψ_{05}	0.130	-0.290	0.811
θ_9	-0.008	-0.022	-0.000	ψ_{06}	0.618	-0.278	2.400
ρ	0.785	0.650	0.929	ψ_{07}	0.265	-0.093	0.742
σ	0.314	0.289	0.344	ψ_{08}	0.016	-0.006	0.053
				ψ_{09}	0.001	-0.020	0.023

Notes: “2.5%” and “97.5%” indicate the percentiles of MCMC samples.

Table S4.2: Estimates of parameters in the regression model with no weather variable among explanatory variables (i.e., no $D_{AH,t}$ in Eq (7))

	Posterior mean	2.5%	97.5%		Posterior mean	2.5%	97.5%
α_0	-0.885	-1.662	-0.183	ψ_{11}	0.175	-0.055	0.674
α_1	0.536	0.026	1.416	ψ_{12}	5.609	-0.394	17.485
β_0	-1.909	-4.244	0.090	ψ_{13}	0.999	-1.775	5.701
β_1	-1.010	-4.273	2.006	ψ_{14}	7.584	0.386	17.236
β_2	-0.298	-6.682	5.640	ψ_{15}	3.085	-0.010	9.731
γ_1	0.038	0.001	0.132	ψ_{16}	3.167	0.051	8.405
γ_2	0.517	0.012	1.826	ψ_{17}	0.088	-0.185	0.539
γ_3	1.057	0.064	2.674	ψ_{18}	0.023	-0.006	0.067
γ_4	0.185	0.007	0.591	ψ_{19}	0.028	-0.007	0.069
γ_5	0.094	0.003	0.320	ψ_{21}	0.355	-0.041	1.225
γ_6	0.121	0.004	0.429	ψ_{22}	17.395	0.207	49.026
γ_7	0.118	0.008	0.271	ψ_{23}	11.613	-0.742	32.882
γ_8	0.005	0.000	0.014	ψ_{24}	1.796	-0.180	5.933
γ_9	0.007	0.000	0.022	ψ_{25}	3.653	0.070	10.725
ρ	0.665	0.558	0.771	ψ_{26}	4.296	0.032	13.076
σ	0.301	0.278	0.326	ψ_{27}	0.282	-0.154	1.082
				ψ_{28}	0.027	-0.006	0.096
				ψ_{29}	0.185	0.019	0.393
				ψ_{01}	0.050	-0.093	0.291
				ψ_{02}	0.804	-1.195	4.308
				ψ_{03}	-0.248	-2.087	1.780
				ψ_{04}	0.431	-0.356	1.690
				ψ_{05}	0.133	-0.195	0.715
				ψ_{06}	0.564	-0.204	2.118
				ψ_{07}	0.162	-0.149	0.591
				ψ_{08}	0.020	-0.006	0.057
				ψ_{09}	-0.000	-0.018	0.019

Notes: “2.5%” and “97.5%” indicate the percentiles of MCMC samples.

Table S4.3: Estimates of parameters in the regression model with the nationwide average of absolute humidity substituting $D_{AH,t}$ in Eq (7)

	Posterior mean	2.5%	97.5%		Posterior mean	2.5%	97.5%
α_0	-0.742	-2.043	0.553	ψ_{11}	0.062	-0.227	0.501
α_1	0.757	0.031	2.122	ψ_{12}	3.341	-2.918	14.335
α_2	-0.017	-0.062	-0.000	ψ_{13}	-0.210	-3.758	4.790
β_0	-2.596	-5.499	-0.184	ψ_{14}	12.077	1.991	23.092
β_1	-0.349	-4.615	3.410	ψ_{15}	2.529	-0.563	8.867
β_2	-0.937	-7.118	5.203	ψ_{16}	3.455	-0.189	9.109
γ_1	0.117	0.009	0.332	ψ_{17}	0.016	-0.251	0.379
γ_2	1.798	0.177	5.776	ψ_{18}	0.034	-0.004	0.086
γ_3	2.443	0.429	4.954	ψ_{19}	0.052	-0.010	0.113
γ_4	0.648	0.054	1.571	ψ_{21}	0.285	-0.172	1.194
γ_5	0.492	0.036	1.404	ψ_{22}	15.613	-1.248	46.437
γ_6	0.374	0.037	1.206	ψ_{23}	10.352	-2.196	31.802
γ_7	0.122	0.008	0.333	ψ_{24}	1.485	-0.841	5.939
γ_8	0.006	0.000	0.020	ψ_{25}	3.120	-0.471	9.914
γ_9	0.052	0.014	0.099	ψ_{26}	4.122	-0.204	13.010
θ_1	-0.007	-0.024	-0.000	ψ_{27}	0.335	-0.153	1.175
θ_2	-0.259	-0.648	-0.018	ψ_{28}	0.025	-0.009	0.095
θ_3	-0.039	-0.140	-0.001	ψ_{29}	0.143	-0.029	0.361
θ_4	-0.027	-0.082	-0.001	ψ_{01}	-0.002	-0.258	0.277
θ_5	-0.038	-0.107	-0.002	ψ_{02}	0.444	-3.800	5.442
θ_6	-0.054	-0.133	-0.003	ψ_{03}	-0.778	-3.731	2.677
θ_7	-0.005	-0.017	-0.000	ψ_{04}	0.134	-1.107	1.656
θ_8	-0.000	-0.001	-0.000	ψ_{05}	-0.140	-1.066	0.782
θ_9	-0.006	-0.010	-0.003	ψ_{06}	0.875	-0.514	3.124
ρ	0.715	0.581	0.850	ψ_{07}	0.237	-0.151	0.711
σ	0.306	0.280	0.336	ψ_{08}	0.017	-0.010	0.059
				ψ_{09}	-0.036	-0.081	0.006

Notes: "2.5%" and "97.5%" indicate the percentiles of MCMC samples.

Table S4.4: Estimates of parameters in the regression model with the nationwide average of outside temperature substituting $D_{AH,t}$ in Eq (7)

	Posterior mean	2.5%	97.5%		Posterior mean	2.5%	97.5%
α_0	-0.573	-1.874	0.859	ψ_{11}	0.073	-0.250	0.557
α_1	0.713	0.023	2.123	ψ_{12}	4.371	-3.132	17.806
α_2	-0.014	-0.052	-0.000	ψ_{13}	-0.679	-4.335	4.136
β_0	-3.129	-6.223	-0.621	ψ_{14}	11.181	0.640	22.736
β_1	-0.588	-5.410	3.296	ψ_{15}	2.544	-0.906	9.499
β_2	-1.362	-7.348	4.997	ψ_{16}	3.467	-0.236	9.173
γ_1	0.124	0.007	0.369	ψ_{17}	0.056	-0.236	0.512
γ_2	1.881	0.154	6.100	ψ_{18}	0.032	-0.005	0.086
γ_3	2.811	0.528	5.497	ψ_{19}	0.053	-0.015	0.116
γ_4	0.811	0.085	1.907	ψ_{21}	0.280	-0.198	1.181
γ_5	0.699	0.045	1.982	ψ_{22}	15.084	-1.349	44.539
γ_6	0.430	0.036	1.422	ψ_{23}	10.640	-2.467	32.596
γ_7	0.112	0.007	0.331	ψ_{24}	1.282	-1.170	5.706
γ_8	0.006	0.000	0.020	ψ_{25}	3.067	-0.657	10.060
γ_9	0.055	0.012	0.109	ψ_{26}	4.082	-0.365	13.219
θ_1	-0.006	-0.018	-0.000	ψ_{27}	0.350	-0.161	1.215
θ_2	-0.164	-0.434	-0.010	ψ_{28}	0.025	-0.009	0.093
θ_3	-0.038	-0.133	-0.001	ψ_{29}	0.129	-0.038	0.341
θ_4	-0.022	-0.066	-0.001	ψ_{01}	0.001	-0.285	0.324
θ_5	-0.036	-0.098	-0.002	ψ_{02}	0.383	-4.396	6.007
θ_6	-0.039	-0.099	-0.002	ψ_{03}	-0.715	-4.122	3.010
θ_7	-0.004	-0.013	-0.000	ψ_{04}	0.027	-1.353	1.661
θ_8	-0.000	-0.001	-0.000	ψ_{05}	-0.330	-1.604	0.715
θ_9	-0.004	-0.007	-0.002	ψ_{06}	0.973	-0.618	3.327
ρ	0.736	0.589	0.887	ψ_{07}	0.306	-0.107	0.801
σ	0.308	0.282	0.337	ψ_{08}	0.019	-0.008	0.060
				ψ_{09}	-0.039	-0.091	0.007

Notes: “2.5%” and “97.5%” indicate the percentiles of MCMC samples.

Figure S4.1: Nationwide dummy for outside temperature

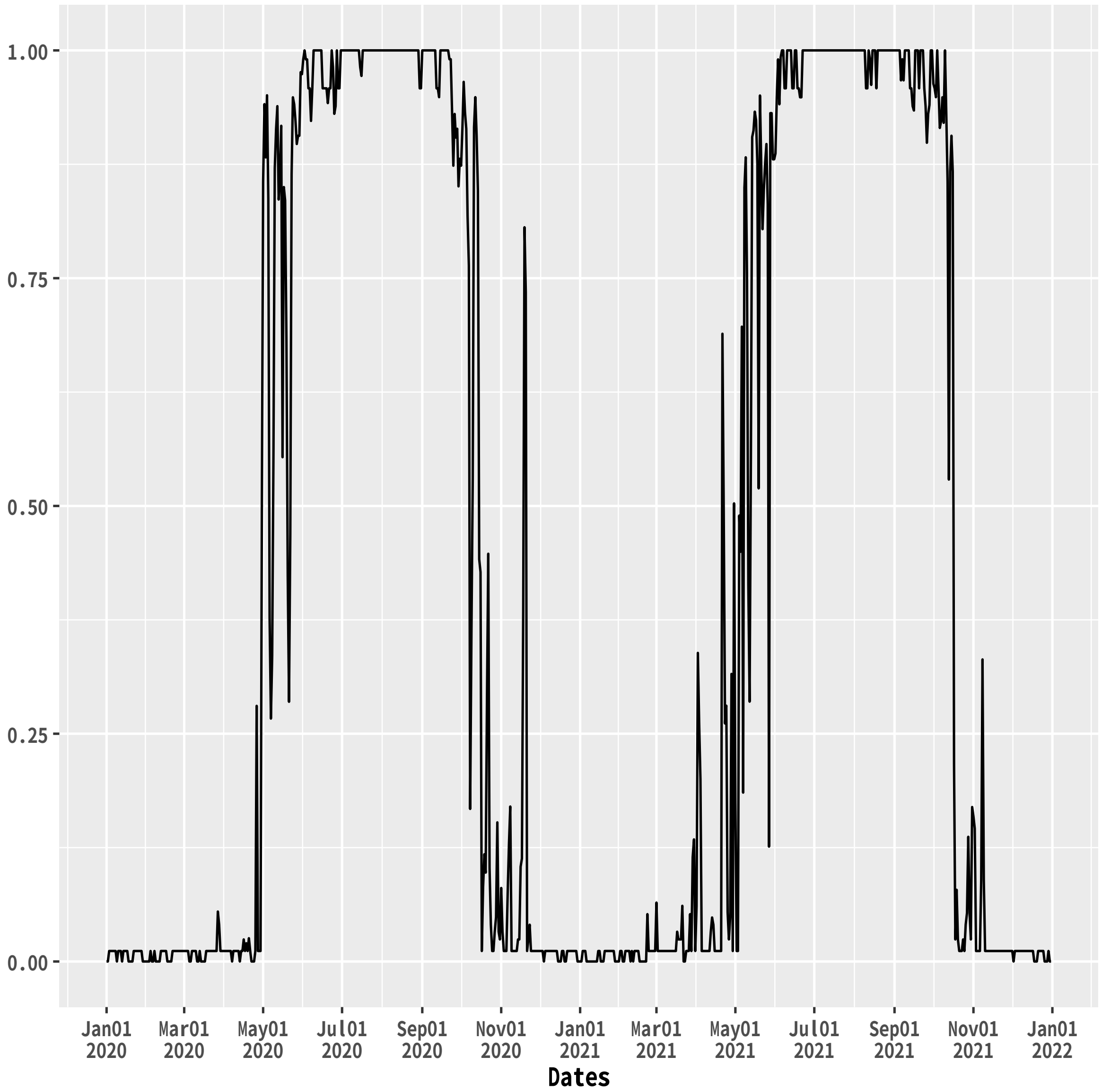

Notes: This series is the population-weighted nationwide average of prefectural dummies for outside temperature being no less than $18°C$ at the capital of each prefecture on each date. The sample period shown in the figure is from January 1, 2020, to December 31, 2021.

Figure S4.2: Nationwide average of absolute humidity

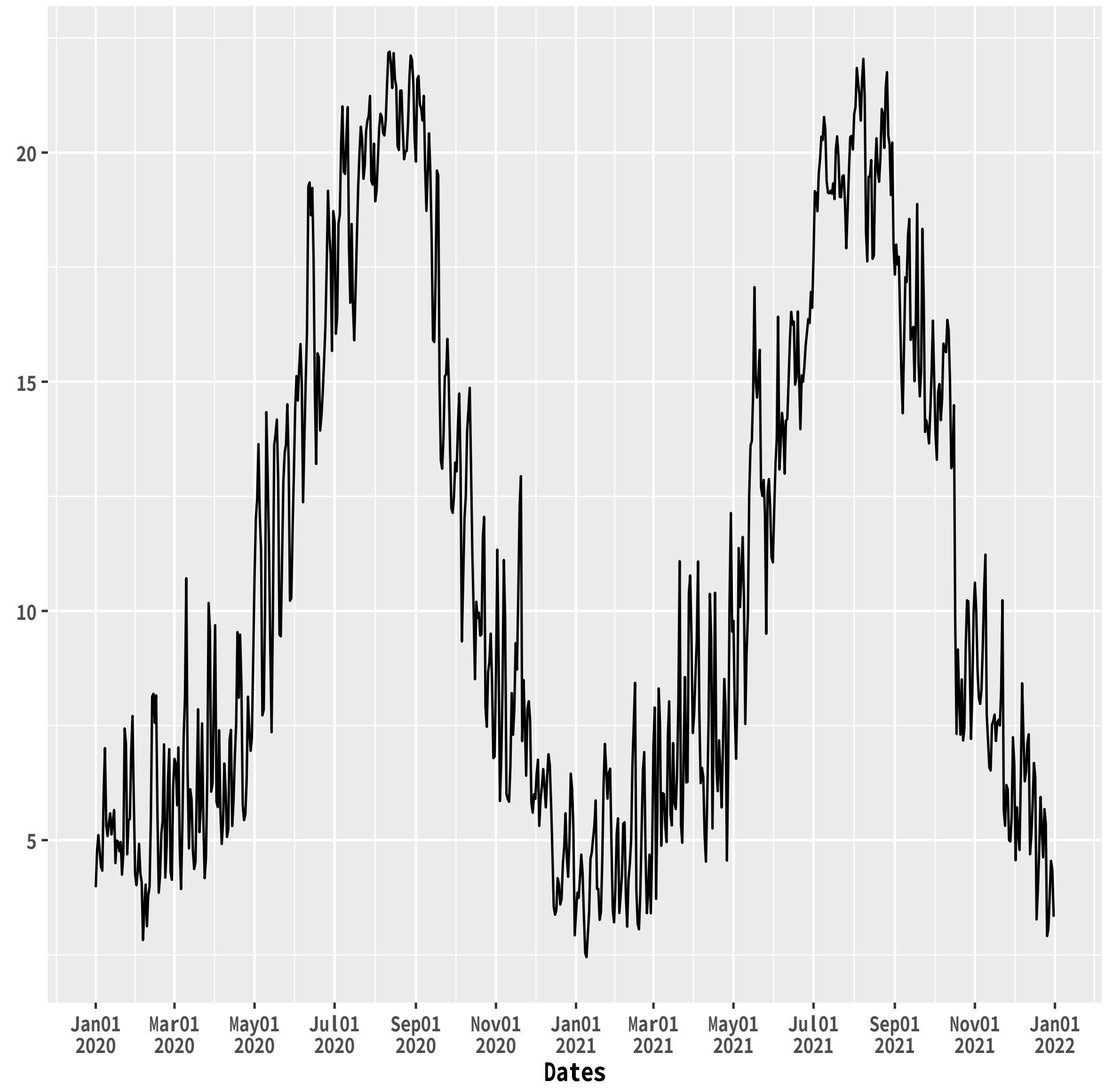

Notes: This series is the population-weighted nationwide average of absolute humidity at the capital of each prefecture on each date. The sample period shown in the figure is from January 1, 2020, to December 31, 2021.

Figure S4.3: Nationwide average of outside temperature

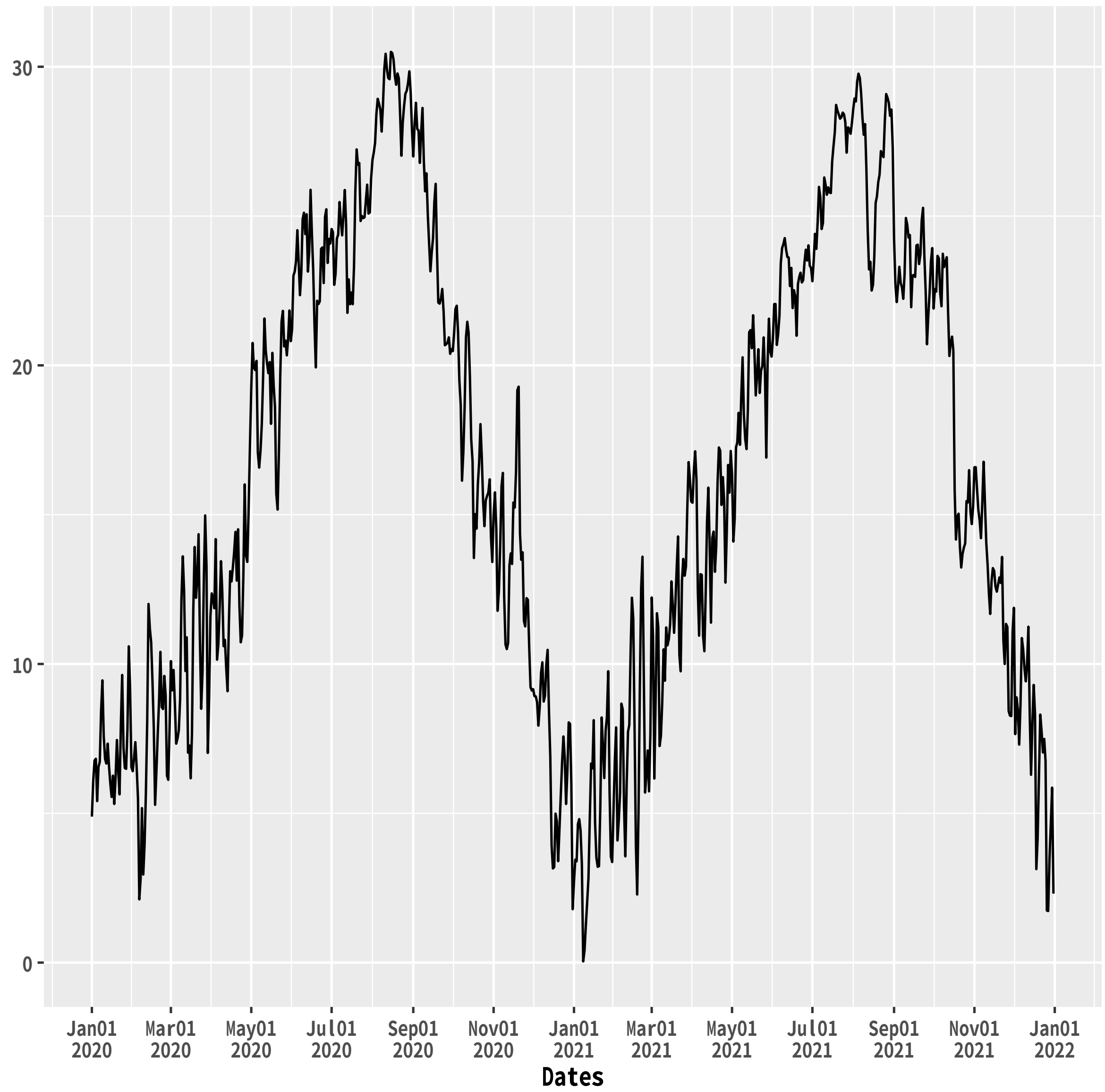

Notes: This series is the population-weighted nationwide average of outside temperature at the capital of each prefecture on each date. The sample period shown in the figure is from January 1, 2020, to December 31, 2021.

Figure S4.4: Fitted values and out-of-sample forecasts of the regression with the nationwide dummy for outside temperature

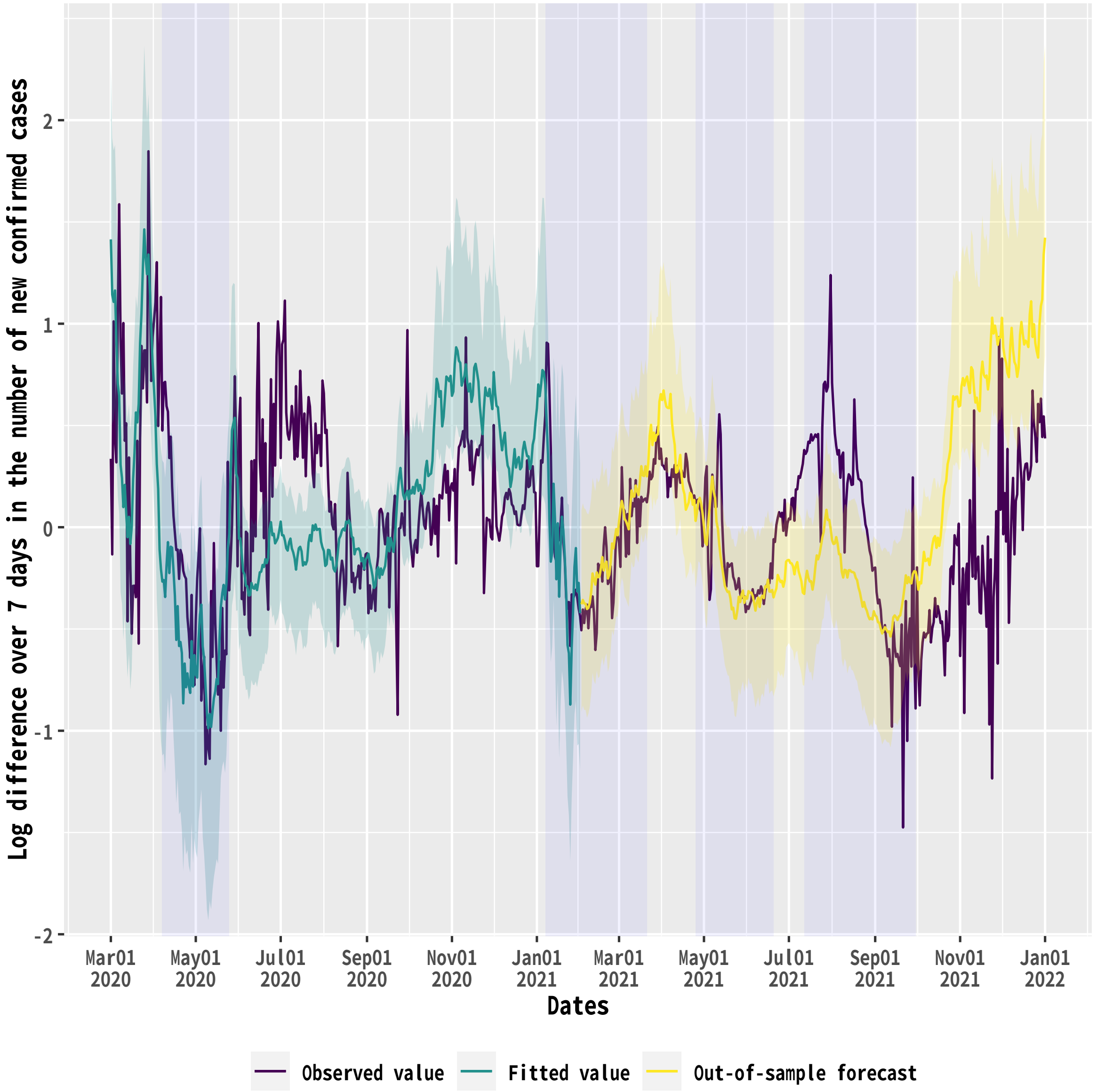

Notes: The dependent variable is the log difference over 7 days in the number of new confirmed cases of COVID-19 in Japan. For out-of-sample forecasts, the time dummy for the second state of emergency is set to zero without changing the posterior means of regression coefficients. The sample period shown in the figure is from March 1, 2020, to January 1, 2022. For the fitted values and the out-of-sample forecasts, the solid line is the posterior mean and the shadowed area indicates the 95% credible interval on each date. Each shadowed period indicates a state of emergency.

Figure S4.5: Fitted values and out-of-sample forecasts of the regression with no weather variable among explanatory variables

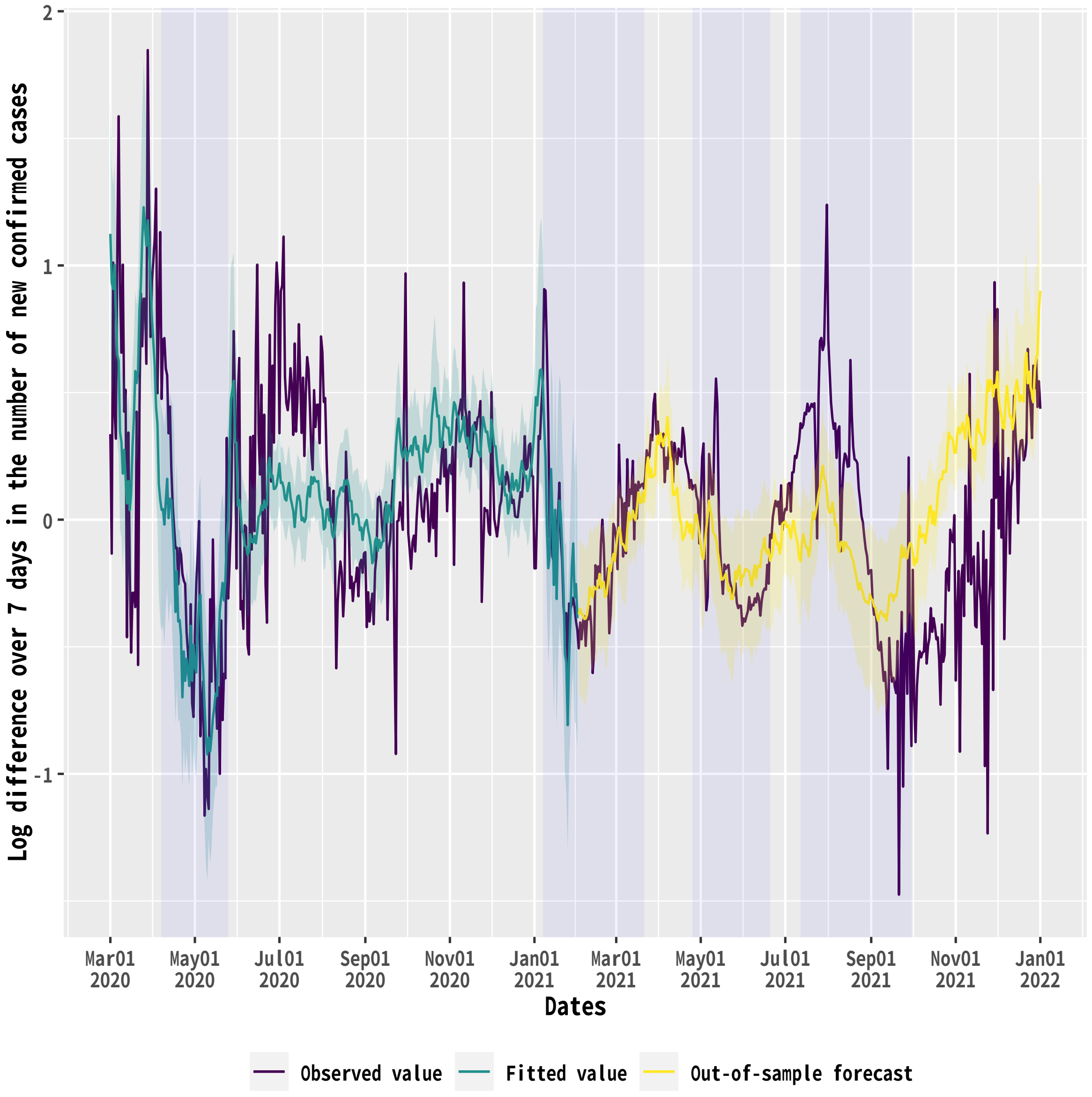

Notes: The dependent variable is the log difference over 7 days in the number of new confirmed cases of COVID-19 in Japan. For out-of-sample forecasts, the time dummy for the second state of emergency is set to zero without changing the posterior means of regression coefficients. The sample period shown in the figure is from March 1, 2020, to January 1, 2022. For the fitted values and the out-of-sample forecasts, the solid line is the posterior mean and the shadowed area indicates the 95% credible interval on each date. Each shadowed period indicates a state of emergency.

Figure S4.6: Fitted values and out-of-sample forecasts of the regression with the nationwide average of absolute humidity

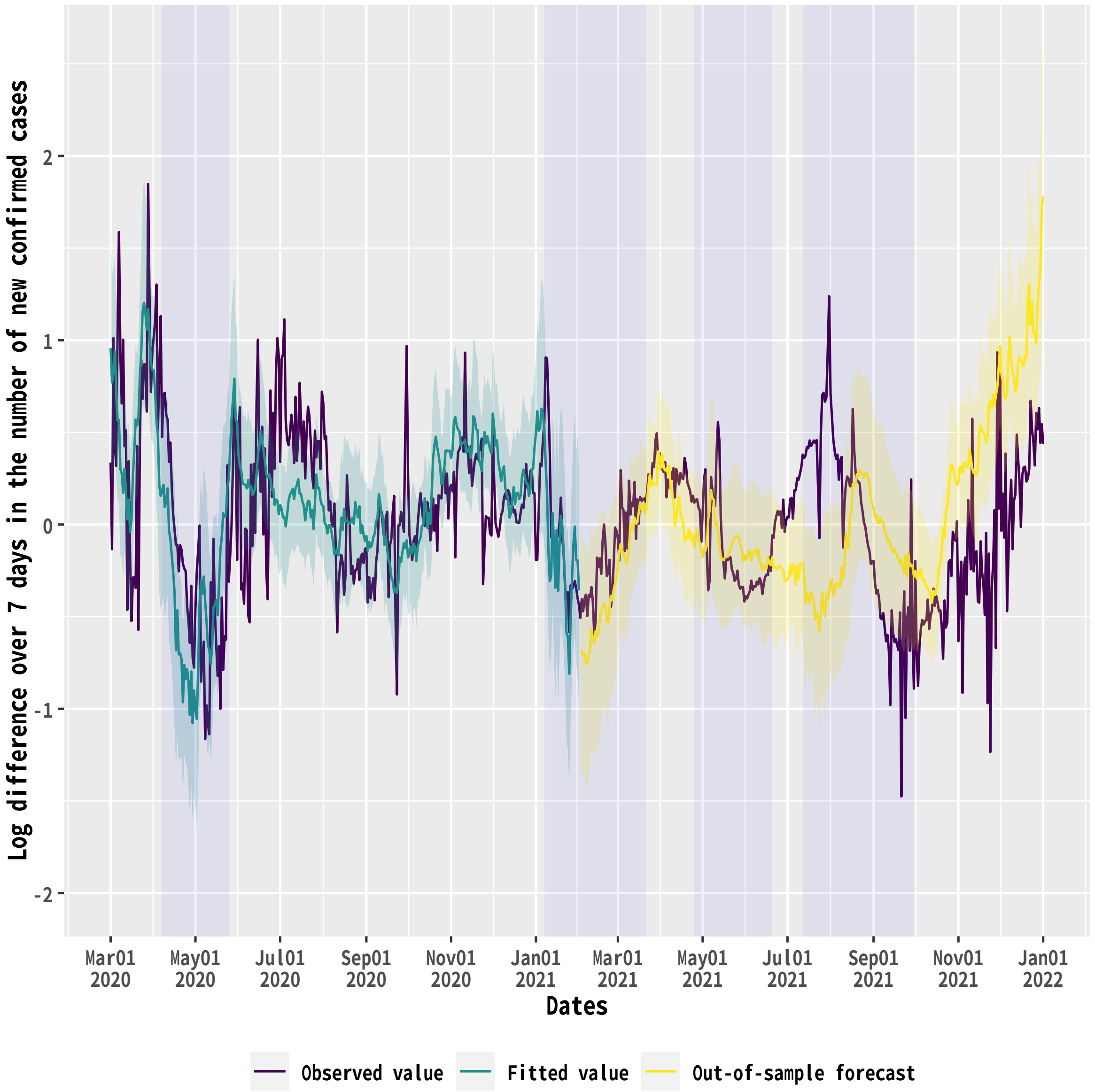

Notes: The dependent variable is the log difference over 7 days in the number of new confirmed cases of COVID-19 in Japan. For out-of-sample forecasts, the time dummy for the second state of emergency is set to zero without changing the posterior means of regression coefficients. The sample period shown in the figure is from March 1, 2020, to January 1, 2022. For the fitted values and the out-of-sample forecasts, the solid line is the posterior mean and the shadowed area indicates the 95% credible interval on each date. Each shadowed period indicates a state of emergency.

Figure S4.7: Fitted values and out-of-sample forecasts of the regression with the nationwide average of outside temperature

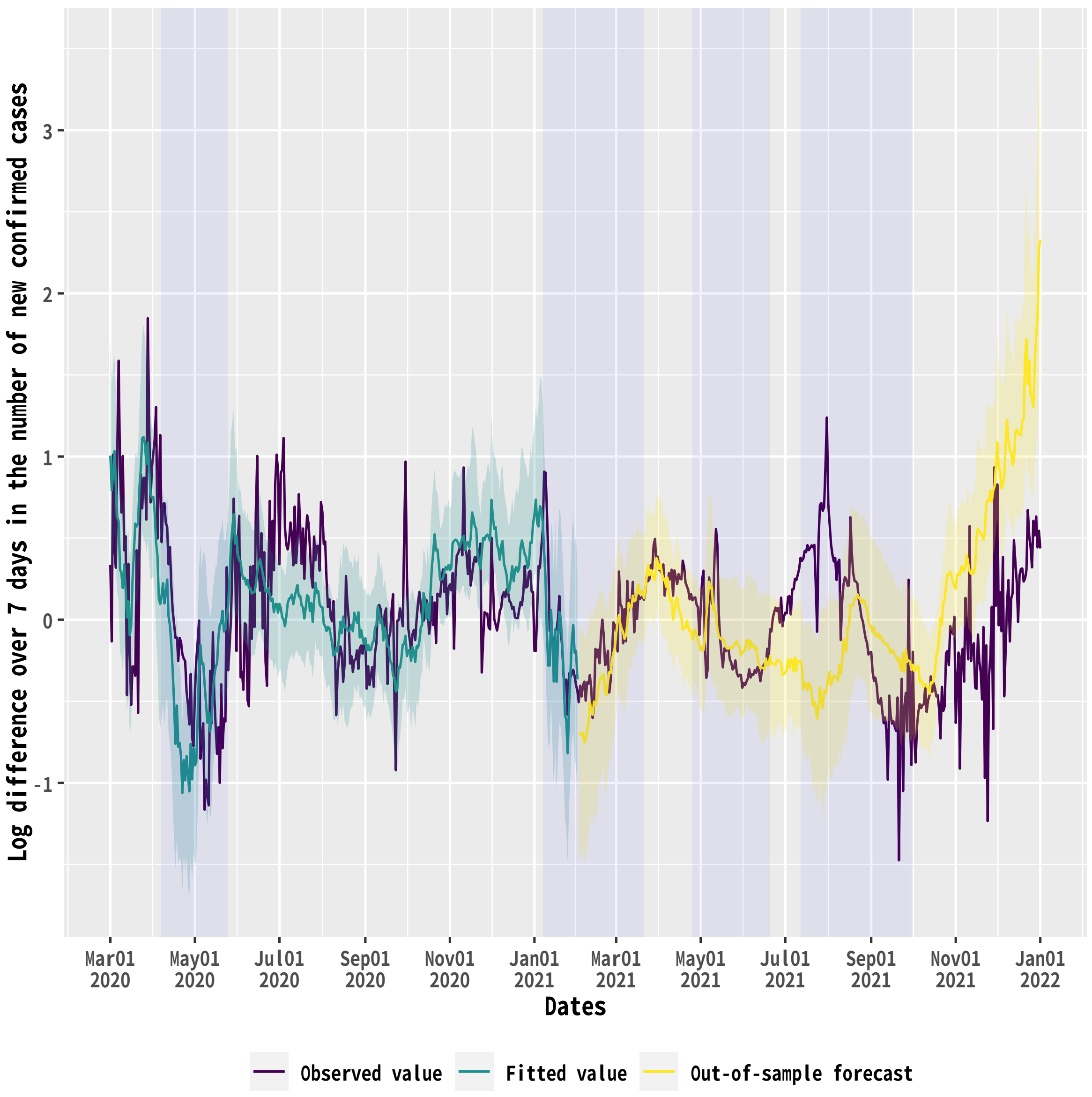

Notes: The dependent variable is the log difference over 7 days in the number of new confirmed cases of COVID-19 in Japan. For out-of-sample forecasts, the time dummy for the second state of emergency is set to zero without changing the posterior means of regression coefficients. The sample period shown in the figure is from March 1, 2020, to January 1, 2022. For the fitted values and the out-of-sample forecasts, the solid line is the posterior mean and the shadowed area indicates the 95% credible interval on each date. Each shadowed period indicates a state of emergency.

Figure S4.8: Decomposition of out-of-sample forecasts of the regression with the nationwide dummy for outside temperature

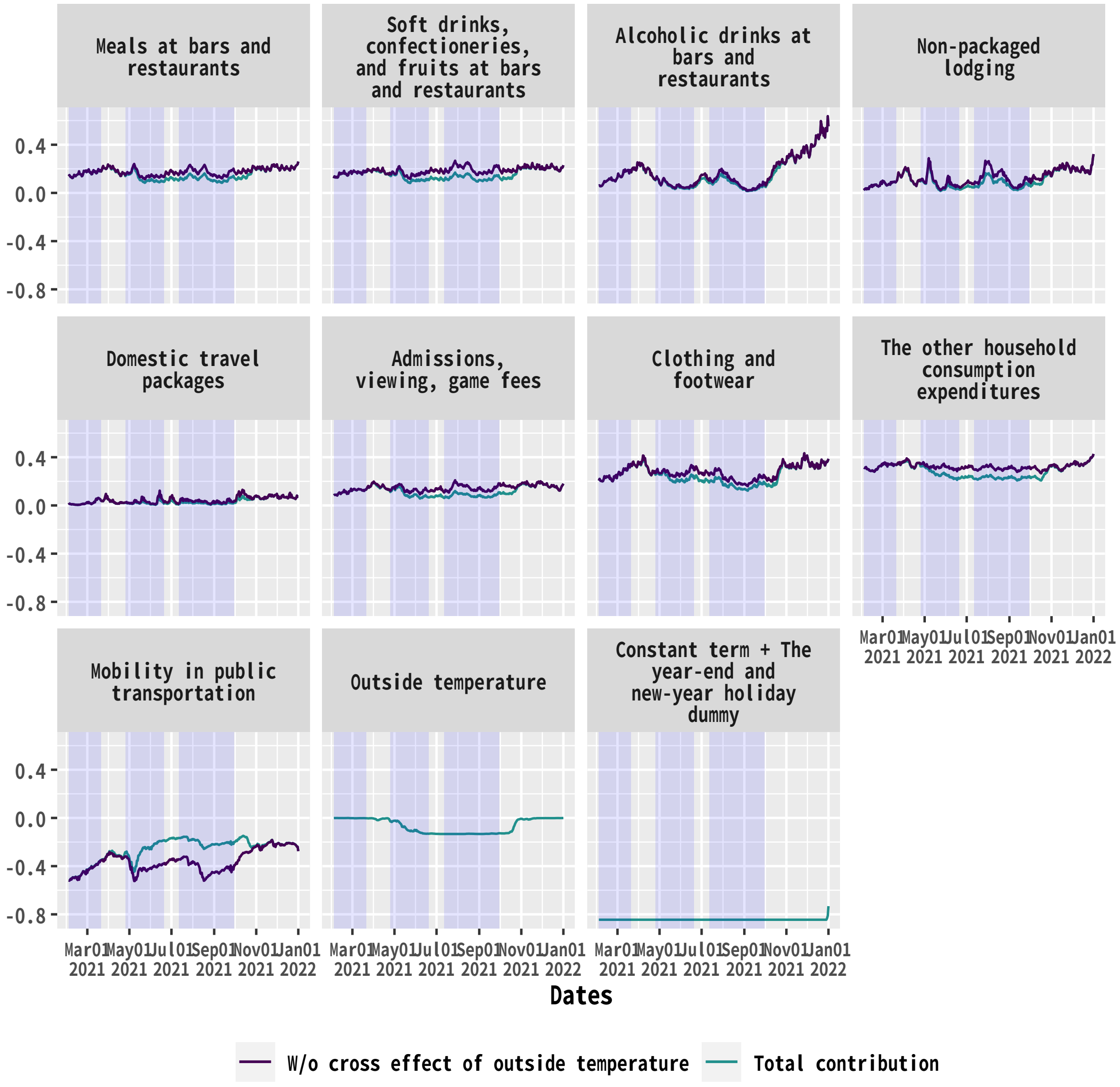

Notes: Each panel shows the product of an explanatory variable and the posterior mean of the corresponding regression coefficient. For out-of-sample forecasts, the time dummy for the second state of emergency is set to zero without changing the posterior means of regression coefficients. The sample period shown in the figure is from February 2, 2021, to January 1, 2022. For household expenditures and mobility in public transportation, "W/o cross effect of outside temperature' indicates the posterior mean of $\gamma_j F(X_{j,t})$ in Eq (7), whereas "Total contribution" indicates the posterior mean of $\gamma_j F(X_{j,t}) + \theta_j F(D_{AH,t} X_{j,t})$ in Eq (7) on each date. Each shadowed period indicates a state of emergency.

Figure S4.9: Decomposition of out-of-sample forecasts of the regression with no weather variable among explanatory variables

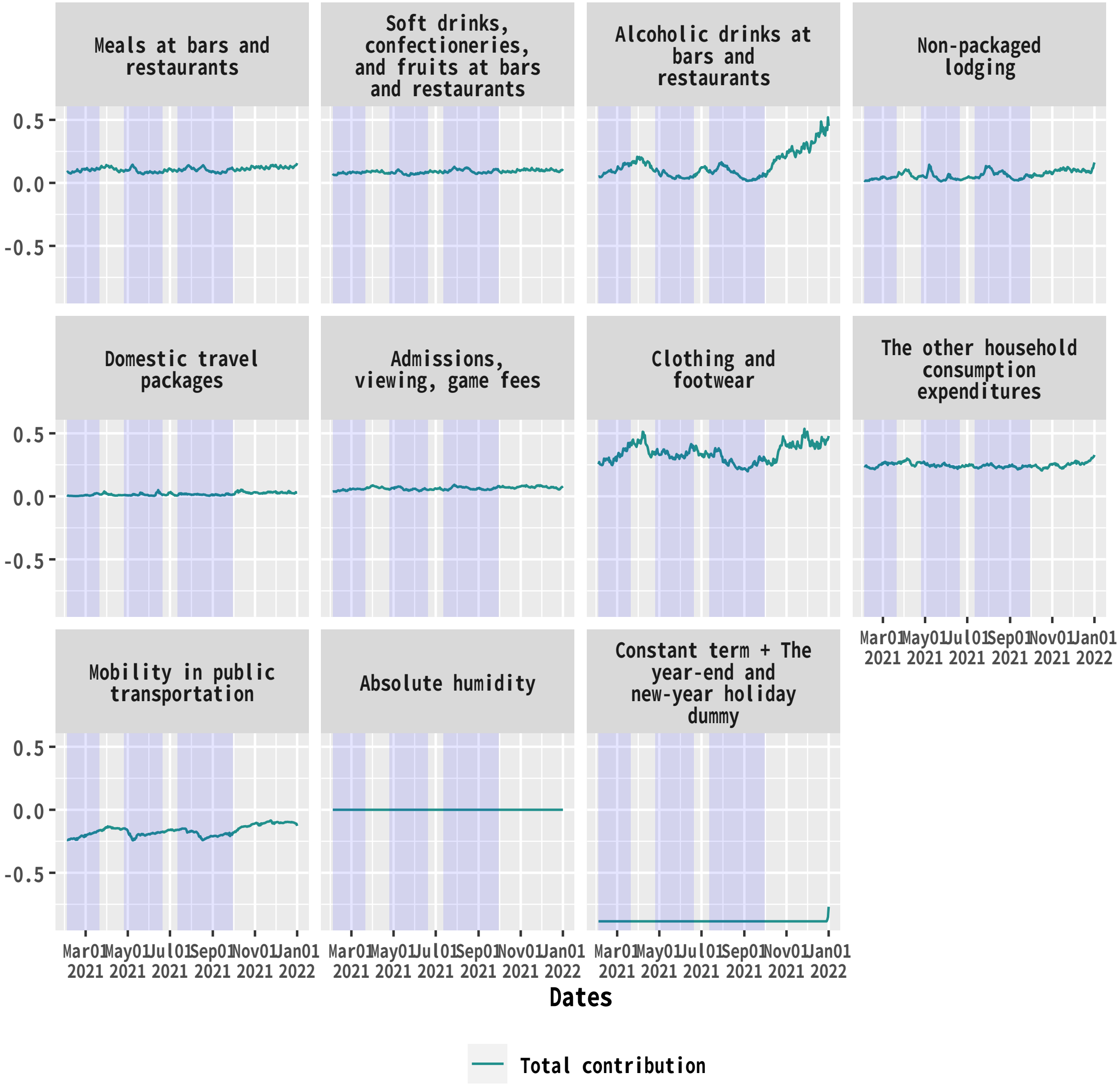

Notes: Each panel shows the product of an explanatory variable and the posterior mean of the corresponding regression coefficient. For out-of-sample forecasts, the time dummy for the second state of emergency is set to zero without changing the posterior means of regression coefficients. The sample period shown in the figure is from February 2, 2021, to January 1, 2022. For household expenditures and mobility in public transportation, "Total contribution" indicates the posterior mean of $\gamma_j F(X_{j,t}))$ in Eq (7) on each date, given $D_{AH,t} = 0$. Each shadowed period indicates a state of emergency.

Figure S4.10: Decomposition of out-of-sample forecasts of the regression with the nationwide average of absolute humidity

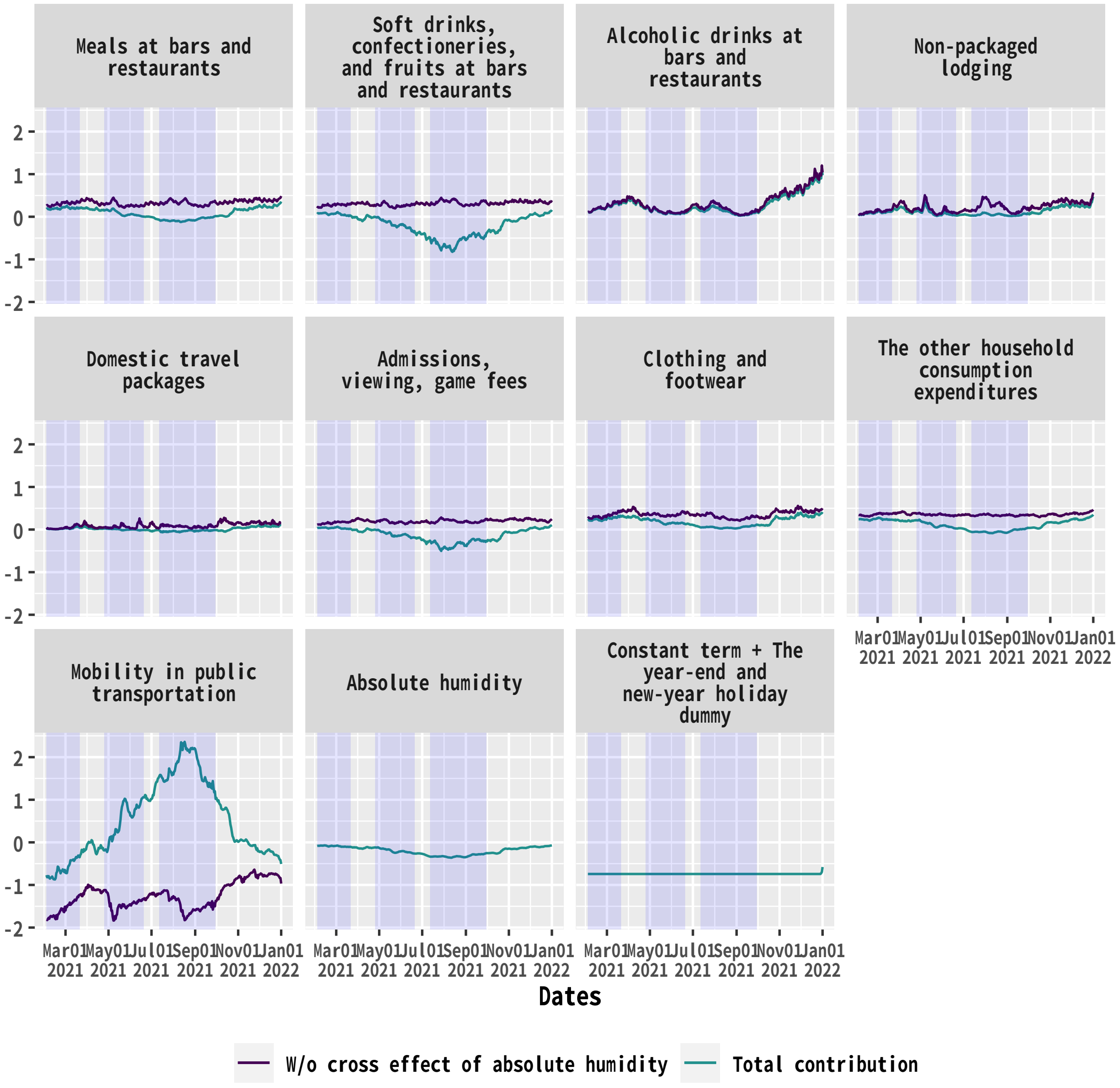

Notes: Each panel shows the product of an explanatory variable and the posterior mean of the corresponding regression coefficient. For out-of-sample forecasts, the time dummy for the second state of emergency is set to zero without changing the posterior means of regression coefficients. The sample period shown in the figure is from February 2, 2021, to January 1, 2022. For household expenditures and mobility in public transportation, "W/o cross effect of absolute humidity' indicates the posterior mean of $\gamma_j F(X_{j,t})$ in Eq (7), whereas "Total contribution" indicates the posterior mean of $\gamma_j F(X_{j,t}) + \theta_j F(D_{AH,t} X_{j,t})$ in Eq (7) on each date. Each shadowed period indicates a state of emergency.

Figure S4.11: Decomposition of out-of-sample forecasts of the regression with the nationwide average of outside temperature

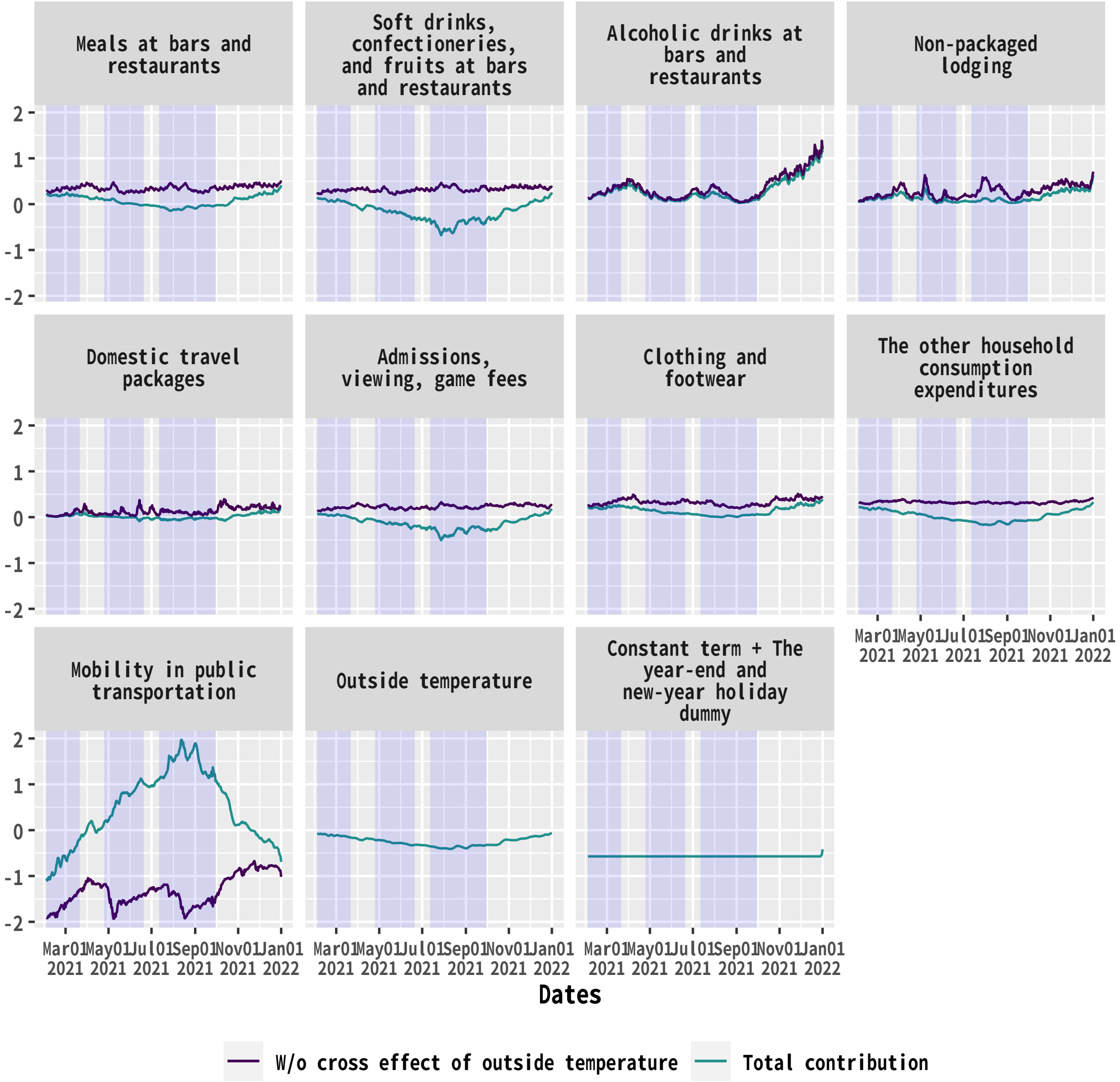

Notes: Each panel shows the product of an explanatory variable and the posterior mean of the corresponding regression coefficient. For out-of-sample forecasts, the time dummy for the second state of emergency is set to zero without changing the posterior means of regression coefficients. The sample period shown in the figure is from February 2, 2021, to January 1, 2022. For household expenditures and mobility in public transportation, "W/o cross effect of outside temperature' indicates the posterior mean of $\gamma_j F(X_{j,t})$ in Eq (7), whereas "Total contribution" indicates the posterior mean of $\gamma_j F(X_{j,t}) + \theta_j F(D_{AH,t} X_{j,t})$ in Eq (7) on each date. Each shadowed period indicates a state of emergency.

Figure S4.12: Decomposition of fitted values of the regression with the nationwide dummy for outside temperature

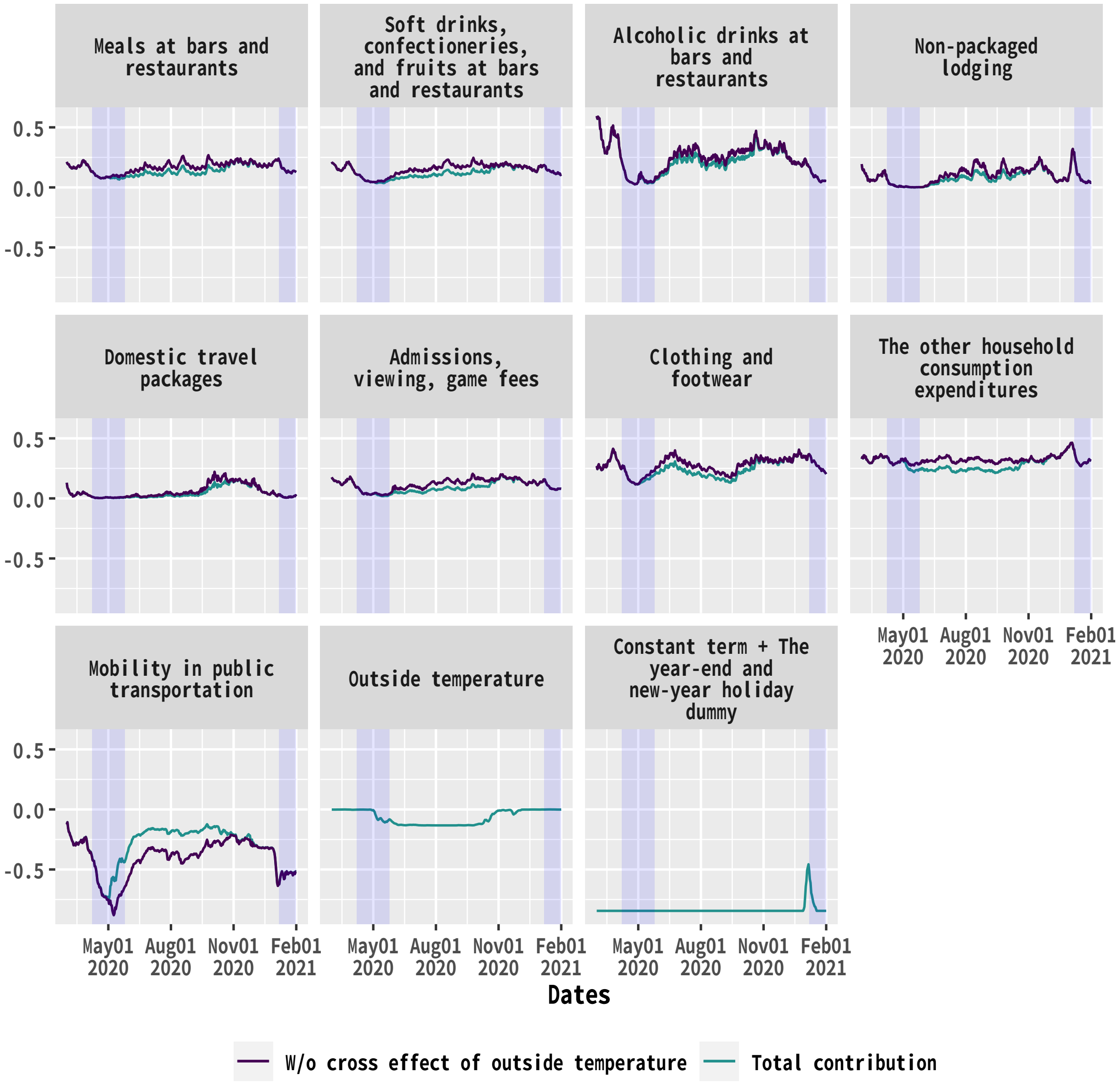

Notes: Each panel shows the product of an explanatory variable and the posterior mean of the corresponding regression coefficient, when time dummies for the period before the first state of emergency, the first state of emergency, and the second state of emergency are set to zero without changing the posterior means of regression coefficients. The sample period shown in the figure is from March 1, 2020, to February 1, 2021. For household expenditures and mobility in public transportation, "W/o cross effect of outside temperature" indicates the posterior mean of $\gamma_j F(X_{j,t})$ in Eq (7), whereas "Total contribution" indicates the posterior mean of $\gamma_j F(X_{j,t}) + \theta_j F(D_{AH,t} X_{j,t})$ in Eq (7) on each date. Each shadowed period indicates a state of emergency.

Figure S4.13: Decomposition of fitted values of the regression with no weather variable among explanatory variables

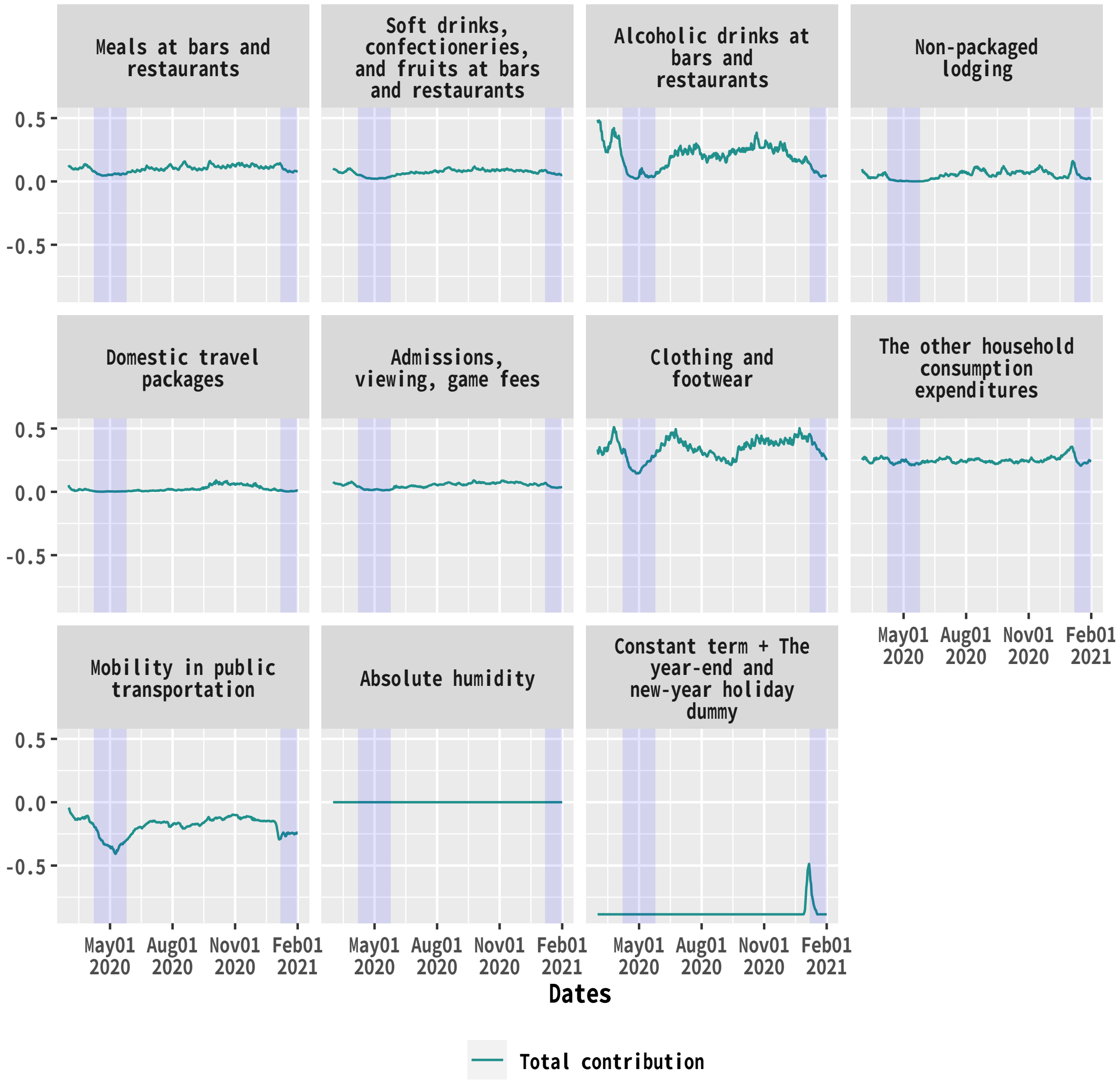

Notes: Each panel shows the product of an explanatory variable and the posterior mean of the corresponding regression coefficient, when time dummies for the period before the first state of emergency, the first state of emergency, and the second state of emergency are set to zero without changing the posterior means of regression coefficients. The sample period shown in the figure is from March 1, 2020, to February 1, 2021. For household expenditures and mobility in public transportation, "Total contribution" indicates the posterior mean of $\gamma_j F(X_{j,t})$ in Eq (7) on each date, given $D_{AH,t} = 0$. Each shadowed period indicates a state of emergency.

Figure S4.14: Decomposition of fitted values of the regression with the nationwide average of absolute humidity

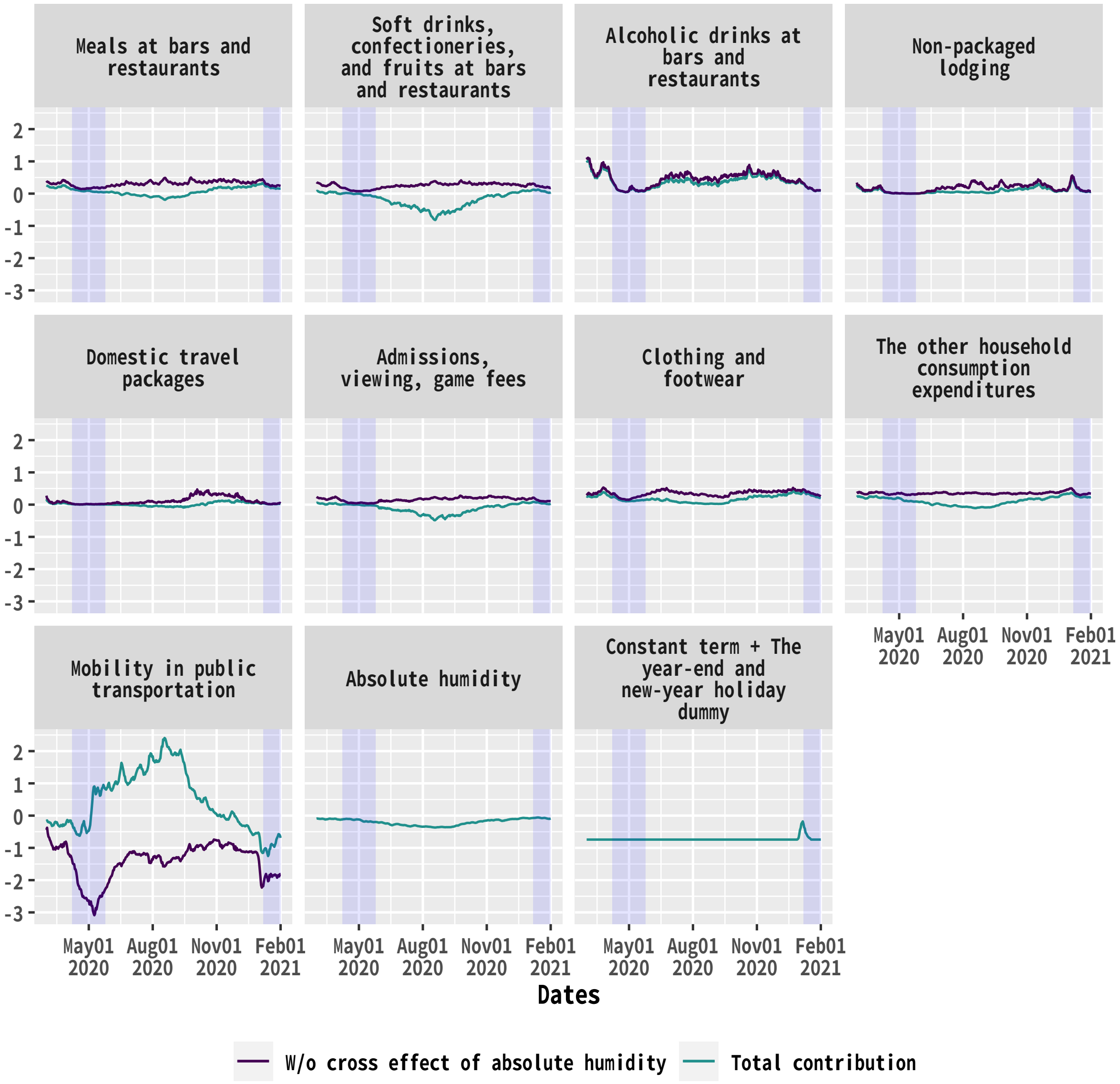

Notes: Each panel shows the product of an explanatory variable and the posterior mean of the corresponding regression coefficient, when time dummies for the period before the first state of emergency, the first state of emergency, and the second state of emergency are set to zero without changing the posterior means of regression coefficients. The sample period shown in the figure is from March 1, 2020, to February 1, 2021. For household expenditures and mobility in public transportation, "W/o cross effect of absolute humidity" indicates the posterior mean of $\gamma_j F(X_{j,t})$ in Eq (7), whereas "Total contribution" indicates the posterior mean of $\gamma_j F(X_{j,t}) + \theta_j F(D_{AH,t} X_{j,t})$ in Eq (7) on each date. Each shadowed period indicates a state of emergency.

Figure S4.15: Decomposition of fitted values of the regression with the nationwide average of outside temperature

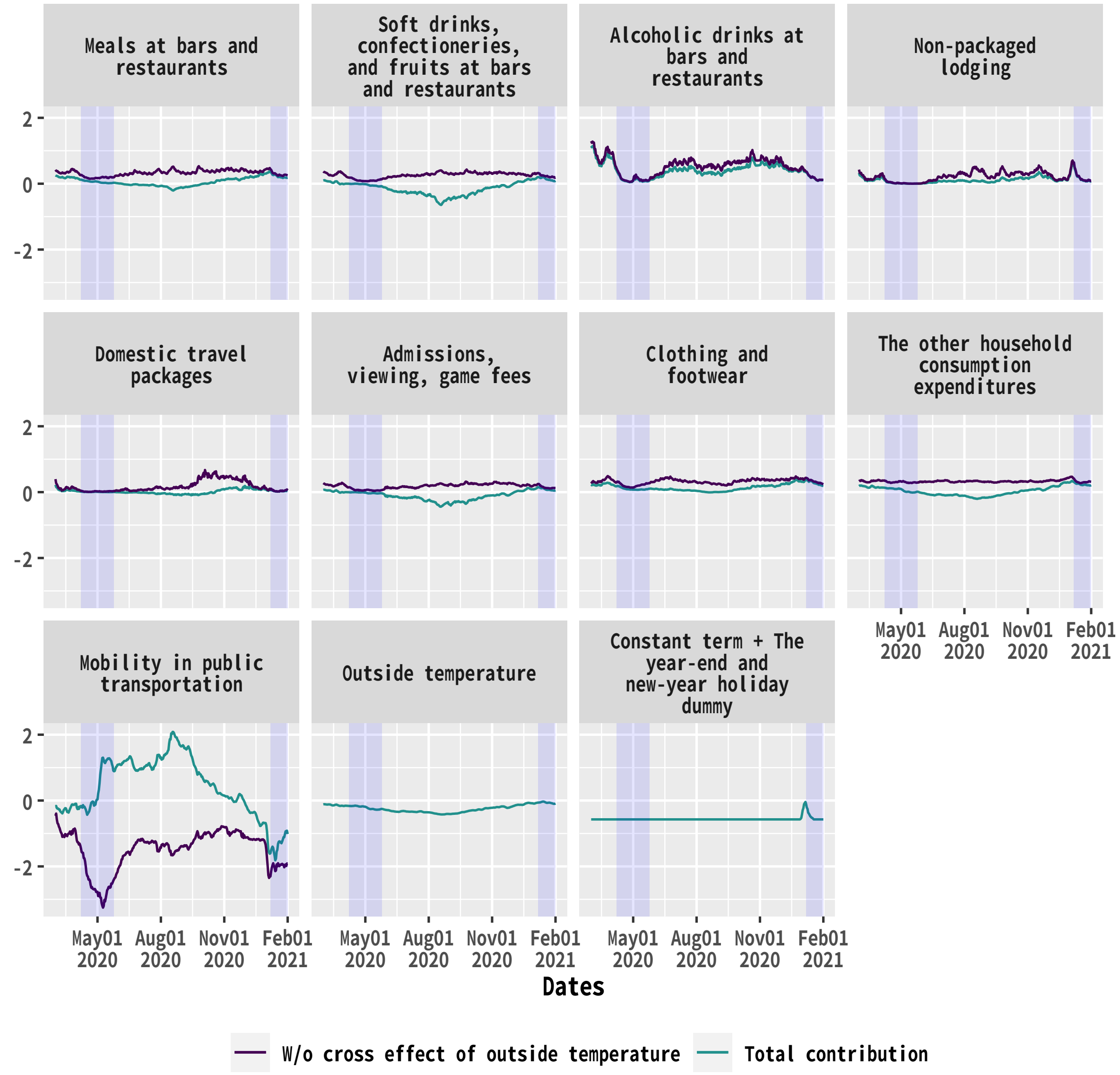

Notes: Each panel shows the product of an explanatory variable and the posterior mean of the corresponding regression coefficient, when time dummies for the period before the first state of emergency, the first state of emergency, and the second state of emergency are set to zero without changing the posterior means of regression coefficients. The sample period shown in the figure is from March 1, 2020, to February 1, 2021. For household expenditures and mobility in public transportation, "W/o cross effect of outside temperature" indicates the posterior mean of $\gamma_j F(X_{j,t})$ in Eq (7), whereas "Total contribution" indicates the posterior mean of $\gamma_j F(X_{j,t}) + \theta_j F(D_{AH,t} X_{j,t})$ in Eq (7) on each date. Each shadowed period indicates a state of emergency.
