# Supplementary material for: Associations between components of household expenditures and the rate of change in the number of new confirmed cases of COVID-19 in Japan: Time-series analysis
Source: PLoS One. 2022 Apr 14;17(4):e0266963. doi: 10.1371/journal.pone.0266963 (PMC9009719; doi:10.1371/journal.pone.0266963)
Supplement: S5 Appendix — (PDF) [file pone.0266963.s007.pdf]

S5 Appendix Sensitivity analysis with offline household expenditures on clothing and footwear for an alternative explanatory variable.

1. Construction of offline household expenditures on clothing and footwear for an explanatory variable

The monthly values of online purchases of selected items per household are available from the Survey of Household Economy published by the Ministry of Internal Affairs and Communications of the Japanese government [57]. These items consist of gifts; food; home electronic appliances; furniture; clothing and footwear; healthcare products and pharmaceuticals; cosmetics; car accessories; physical books; storage devices containing music, videos, and software; digital contents (e.g., electronic books and downloaded music, videos, and software); insurances; lodging, transportation, and packaged travels; tickets; and the other items. The online purchase value of clothing and footwear per household in each month is obtained from this dataset, and then divided by the sum of daily household expenditures on clothing and footwear per household in the same month available from the Family Income and Expenditure Survey [45] to compute the online share. See Figure S5.1 for the time series of the online share. The online share is converted into the offline share by subtracting it from one. Then, the daily values of offline household expenditures on clothing and footwear per household are computed by multiplying household expenditures on clothing and footwear per household on each date by the offline share for the same month. To compute the real value of offline household expenditures on clothing and footwear per household, the same CPI index for clothing and footwear as the one described in S1 Appendix is used, because there are no separate CPI indices available for offline and online purchases.

2. Estimation of the regression model with offline household expenditures on clothing and footwear for an explanatory variable

The regression model is estimated using the real value of offline household expenditures on clothing and footwear per household for $X_{7,t}$. Table S5.1 shows the parameter estimates. Figures S5.2 shows the fitted values and out-of-sample forecasts of the regression. Figures S5.3 and S5.4 show the decomposition of out-of-sample forecasts and the fitted values, respectively, into contributions from explanatory variables.

Figure S5.1: Online share of household expenditures on clothing and footwear

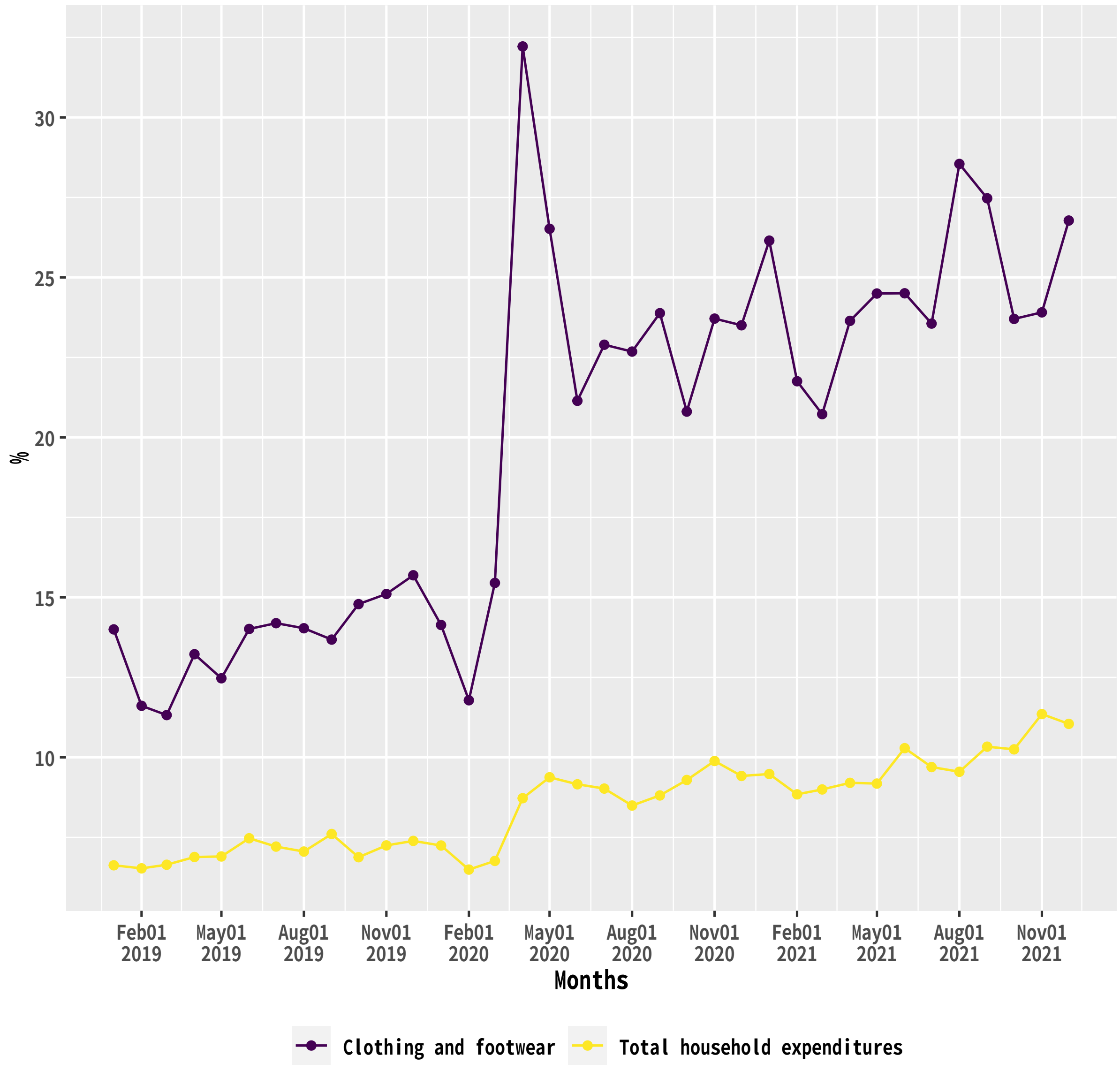

Source: Survey of Household Economy, the Ministry of Internal Affairs and Communications, the Government of Japan [57].

Table S5.1: Estimates of parameters in the regression model when household expenditures on clothing and footwear in the explanatory variables include only offline purchases.

	Posterior mean	2.5%	97.5%		Posterior mean	2.5%	97.5%
α_0	-0.825	-1.665	-0.049	ψ_{11}	0.180	-0.072	0.706
α_1	0.574	0.022	1.651	ψ_{12}	5.585	-0.736	18.535
α_2	-0.120	-0.452	-0.003	ψ_{13}	1.072	-1.921	5.901
β_0	-2.029	-4.503	0.027	ψ_{14}	7.880	0.415	18.282
β_1	-1.192	-4.570	2.126	ψ_{15}	3.273	0.016	10.406
β_2	-1.149	-7.191	4.826	ψ_{16}	3.149	-0.000	8.665
γ_1	0.062	0.008	0.169	ψ_{17}	0.189	-0.186	0.846
γ_2	1.053	0.130	2.913	ψ_{18}	0.026	-0.005	0.076
γ_3	1.322	0.195	3.087	ψ_{19}	0.025	-0.009	0.072
γ_4	0.362	0.055	0.932	ψ_{21}	0.352	-0.051	1.217
γ_5	0.221	0.025	0.589	ψ_{22}	16.102	-0.078	47.232
γ_6	0.272	0.034	0.769	ψ_{23}	12.582	-0.458	34.853
γ_7	0.131	0.016	0.335	ψ_{24}	1.830	-0.211	6.160
γ_8	0.006	0.001	0.017	ψ_{25}	3.753	0.024	10.643
γ_9	0.019	0.003	0.041	ψ_{26}	4.265	0.017	12.910
θ_1	-0.019	-0.069	-0.000	ψ_{27}	0.428	-0.141	1.477
θ_2	-0.411	-1.531	-0.012	ψ_{28}	0.030	-0.005	0.100
θ_3	-0.243	-0.880	-0.005	ψ_{29}	0.162	0.010	0.364
θ_4	-0.133	-0.452	-0.003	ψ_{01}	0.055	-0.091	0.314
θ_5	-0.097	-0.358	-0.003	ψ_{02}	0.877	-1.463	4.574
θ_6	-0.122	-0.456	-0.003	ψ_{03}	-0.183	-2.168	2.222
θ_7	-0.028	-0.105	-0.001	ψ_{04}	0.280	-0.463	1.424
θ_8	-0.002	-0.006	-0.000	ψ_{05}	0.127	-0.302	0.809
θ_9	-0.012	-0.029	-0.001	ψ_{06}	0.581	-0.262	2.287
ρ	0.761	0.641	0.887	ψ_{07}	0.202	-0.173	0.732
σ	0.313	0.287	0.342	ψ_{08}	0.017	-0.006	0.054
				ψ_{09}	0.000	-0.018	0.020

Notes: "2.5%" and "97.5%" indicate the percentiles of MCMC samples.

Figure S5.2: Fitted values and out-of-sample forecasts of the regression when household expenditures on clothing and footwear in the explanatory variables include only offline purchases.

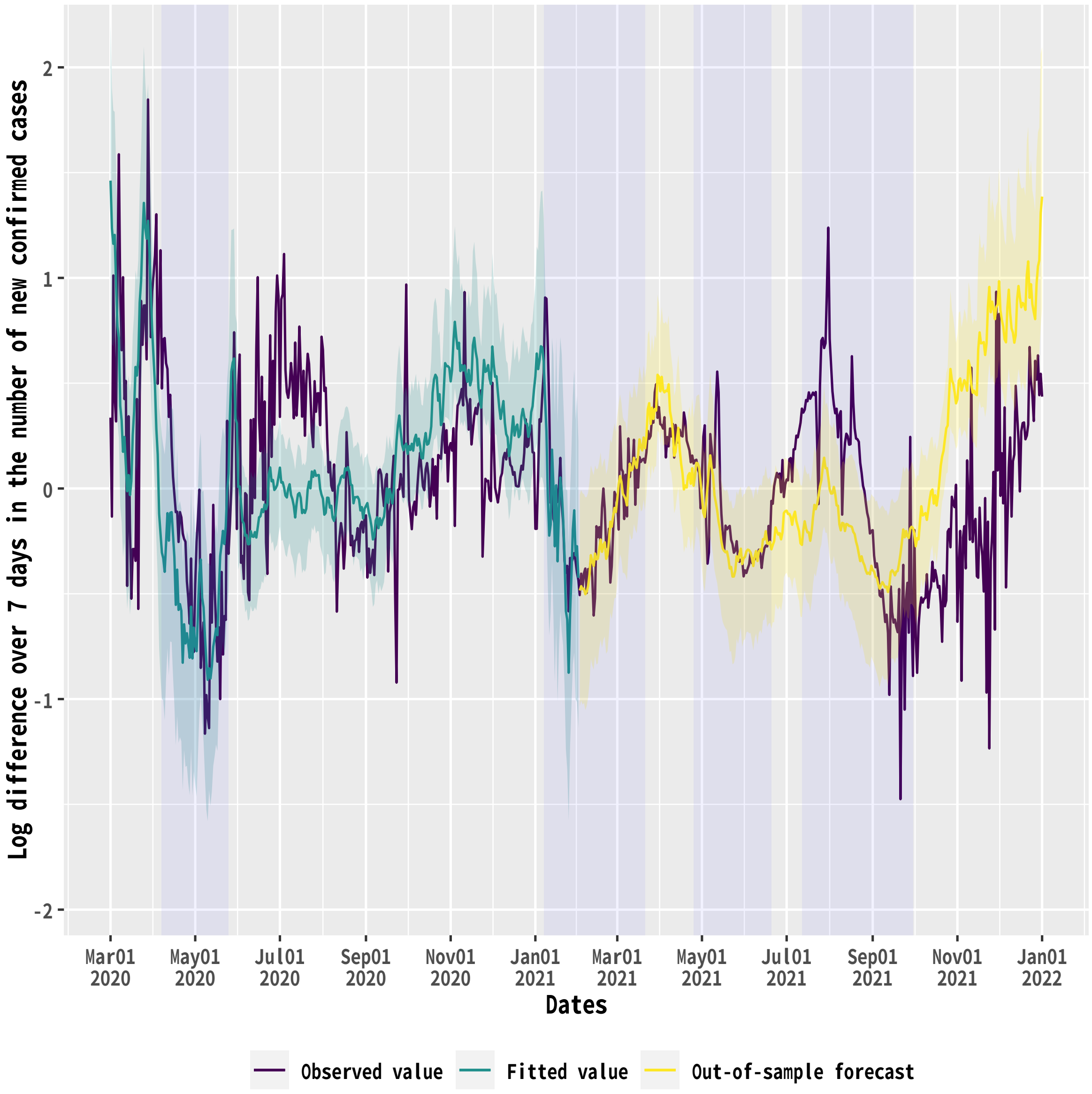

Notes: The dependent variable is the log difference over 7 days in the number of new confirmed cases of COVID-19 in Japan. For out-of-sample forecasts, the time dummy for the second state of emergency is set to zero without changing the posterior means of regression coefficients. The sample period shown in the figure is from March 1, 2020, to January 1, 2022. For the fitted values and the out-of-sample forecasts, the solid line is the posterior mean and the shadowed area indicates the 95% credible interval on each date. Each shadowed period indicates a state of emergency.

Figure S5.3: Decomposition of out-of-sample forecasts of the regression when household expenditures on clothing and footwear in the explanatory variables include only offline purchases.

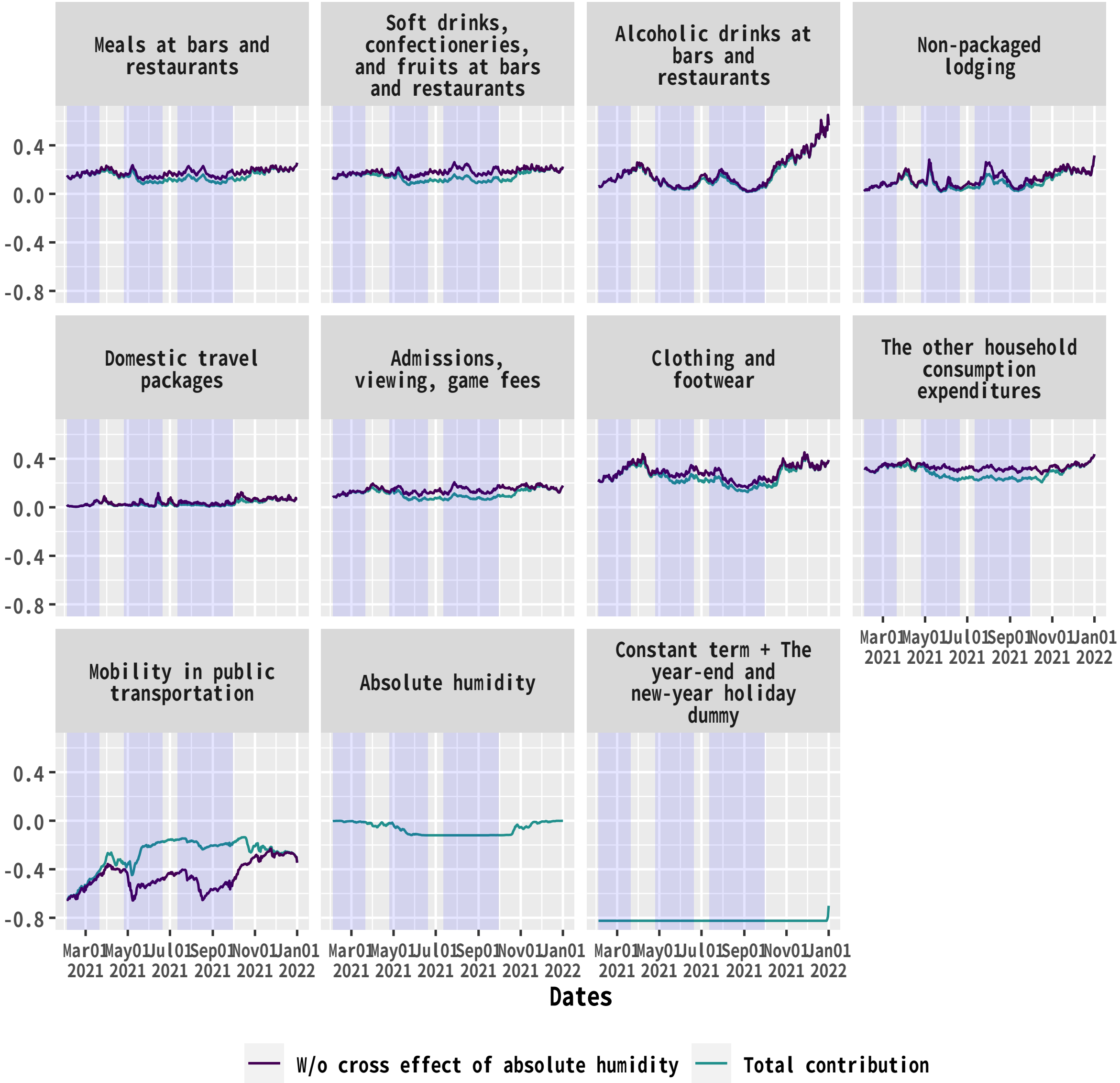

Notes: Each panel shows the product of an explanatory variable and the posterior mean of the corresponding regression coefficient. For out-of-sample forecasts, the time dummy for the second state of emergency is set to zero without changing the posterior means of regression coefficients. The sample period shown in the figure is from February 2, 2021, to January 1, 2022. For household expenditures and mobility in public transportation, "W/o cross effect of absolute humidity' indicates the posterior mean of $\gamma_j F(X_{j,t})$ in Eq (7), whereas "Total contribution" indicates the posterior mean of $\gamma_j F(X_{j,t}) + \theta_j F(D_{AH,t} X_{j,t})$ in Eq (7) on each date. Each shadowed period indicates a state of emergency.

Figure S5.4: Decomposition of fitted values of the regression when household expenditures on clothing and footwear in the explanatory variables include only offline purchases.

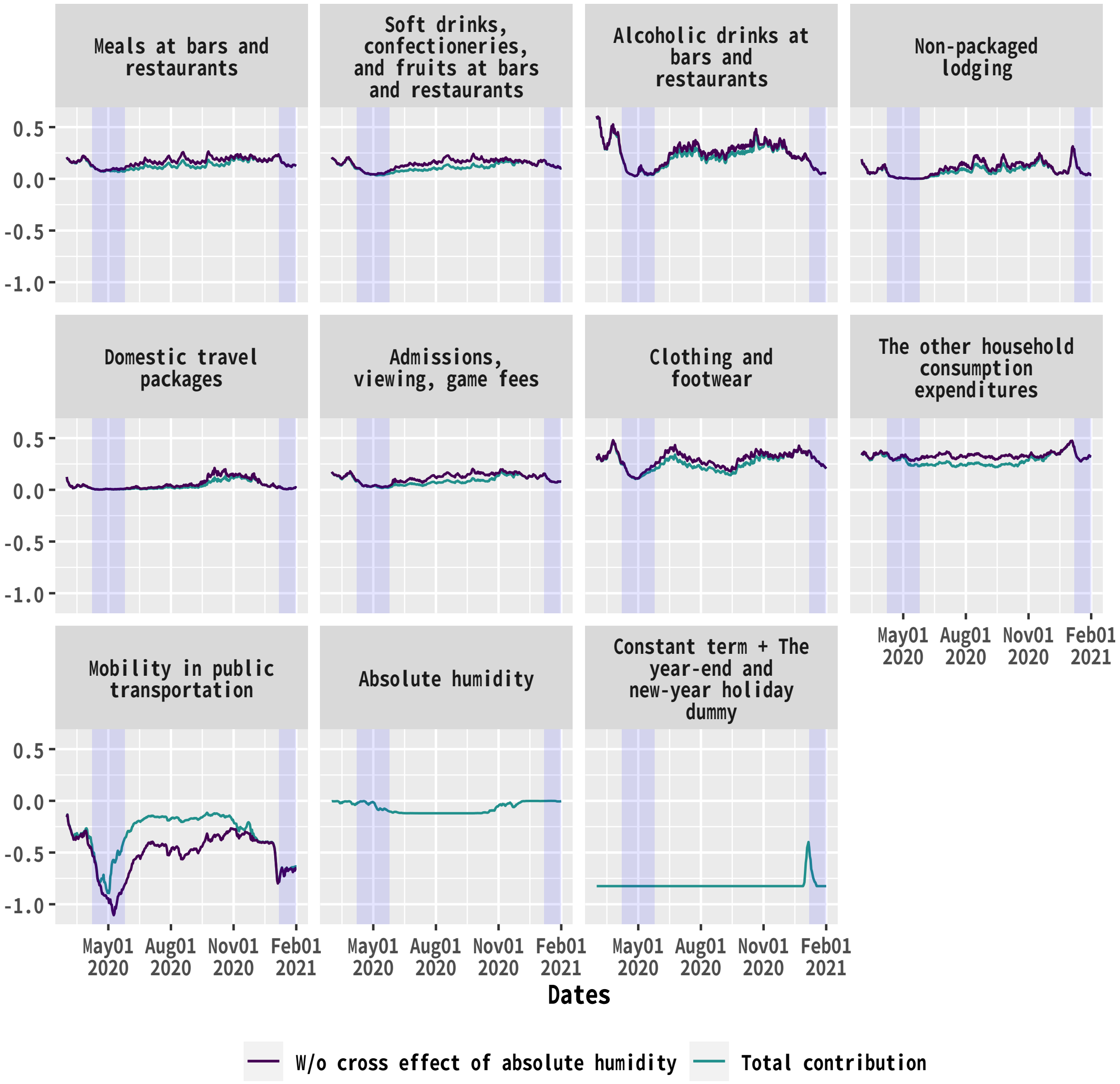

Notes: Each panel shows the product of an explanatory variable and the posterior mean of the corresponding regression coefficient, when time dummies for the period before the first state of emergency, the first state of emergency, and the second state of emergency are set to zero without changing the posterior means of regression coefficients. The sample period shown in the figure is from March 1, 2020, to February 1, 2021. For household expenditures and mobility in public transportation, "W/o cross effect of absolute humidity" indicates the posterior mean of $\gamma_j F(X_{j,t})$ in Eq (7), whereas "Total contribution" indicates the posterior mean of $\gamma_j F(X_{j,t}) + \theta_j F(D_{AH,t} X_{j,t})$ in Eq (7) on each date. Each shadowed period indicates a state of emergency.
