# Supplementary material for: Associations between components of household expenditures and the rate of change in the number of new confirmed cases of COVID-19 in Japan: Time-series analysis
Source: PLoS One. 2022 Apr 14;17(4):e0266963. doi: 10.1371/journal.pone.0266963 (PMC9009719; doi:10.1371/journal.pone.0266963)
Supplement: S1 Fig — (PDF) [file pone.0266963.s008.pdf]

S1 FIG. Nominal values of classified components of household expenditures per household in Japan.

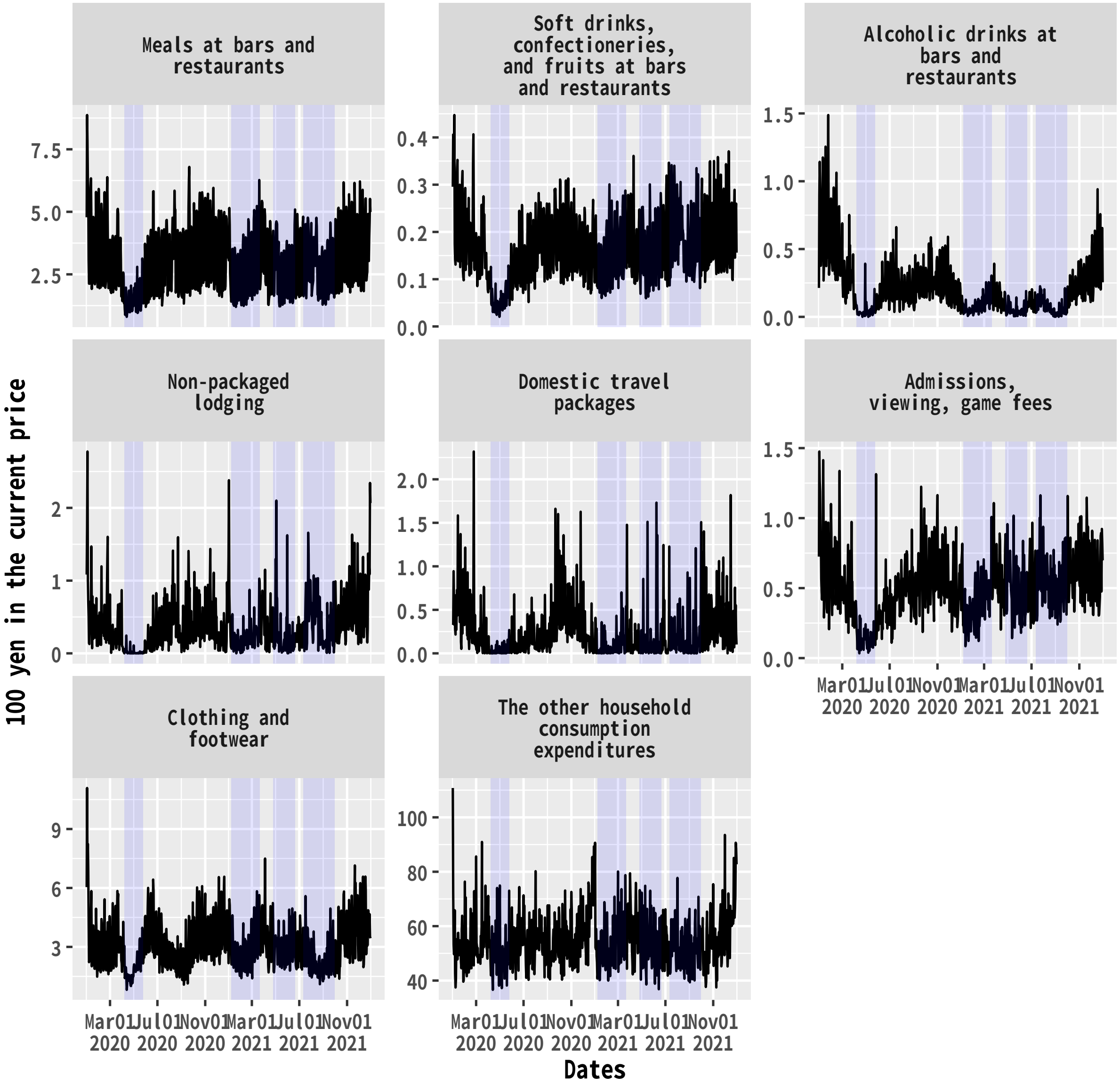

Notes: This figure shows that nominal household expenditures per household for domestic travel packages increased substantially during the Go-To-Travel campaign period between July 22, 2020, and December 27, 2020, while those for non-packaged travels did not. The unit of each series is normalized to 100 yen. "The current price" means the price on each date. The sample period shown in the figure is from January 1, 2020, to December 31, 2021. Each shadowed period indicates a state of emergency.
