# Supplementary material for: Associations between components of household expenditures and the rate of change in the number of new confirmed cases of COVID-19 in Japan: Time-series analysis
Source: PLoS One. 2022 Apr 14;17(4):e0266963. doi: 10.1371/journal.pone.0266963 (PMC9009719; doi:10.1371/journal.pone.0266963)
Supplement: S2 Fig — (PDF) [file pone.0266963.s009.pdf]

S2 FIG. Fitted values and out-of-sample forecasts of the regression with time dummies related to states of emergency.

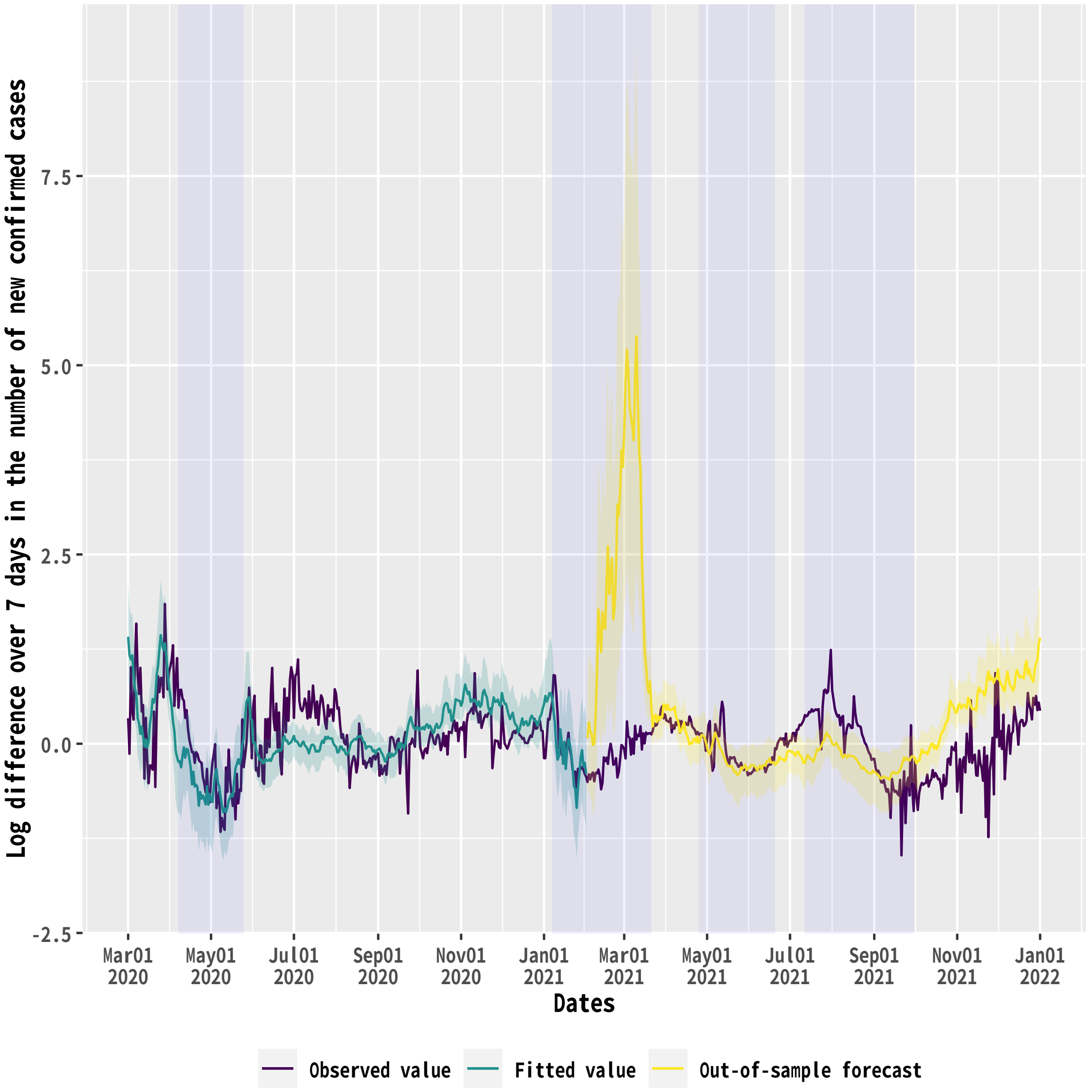

Notes: The dependent variable is the log difference over 7 days in the number of new confirmed cases of COVID-19 in Japan. For out-of-sample forecasts, the time dummy for the second state of emergency is set to one until the end of the second state of emergency. The sample period shown in the figure is from March 1, 2020, to January 1, 2022. For the fitted values and the out-of-sample forecasts, the solid line is the posterior mean and the shadowed area indicates the 95% credible interval on each date. Each shadowed period indicates a state of emergency.
