# Supplementary material for: Associations between components of household expenditures and the rate of change in the number of new confirmed cases of COVID-19 in Japan: Time-series analysis
Source: PLoS One. 2022 Apr 14;17(4):e0266963. doi: 10.1371/journal.pone.0266963 (PMC9009719; doi:10.1371/journal.pone.0266963)
Supplement: S3 Fig — (PDF) [file pone.0266963.s010.pdf]

S3 FIG. Decomposition of fitted values of the regression with time dummies related to states of emergency.

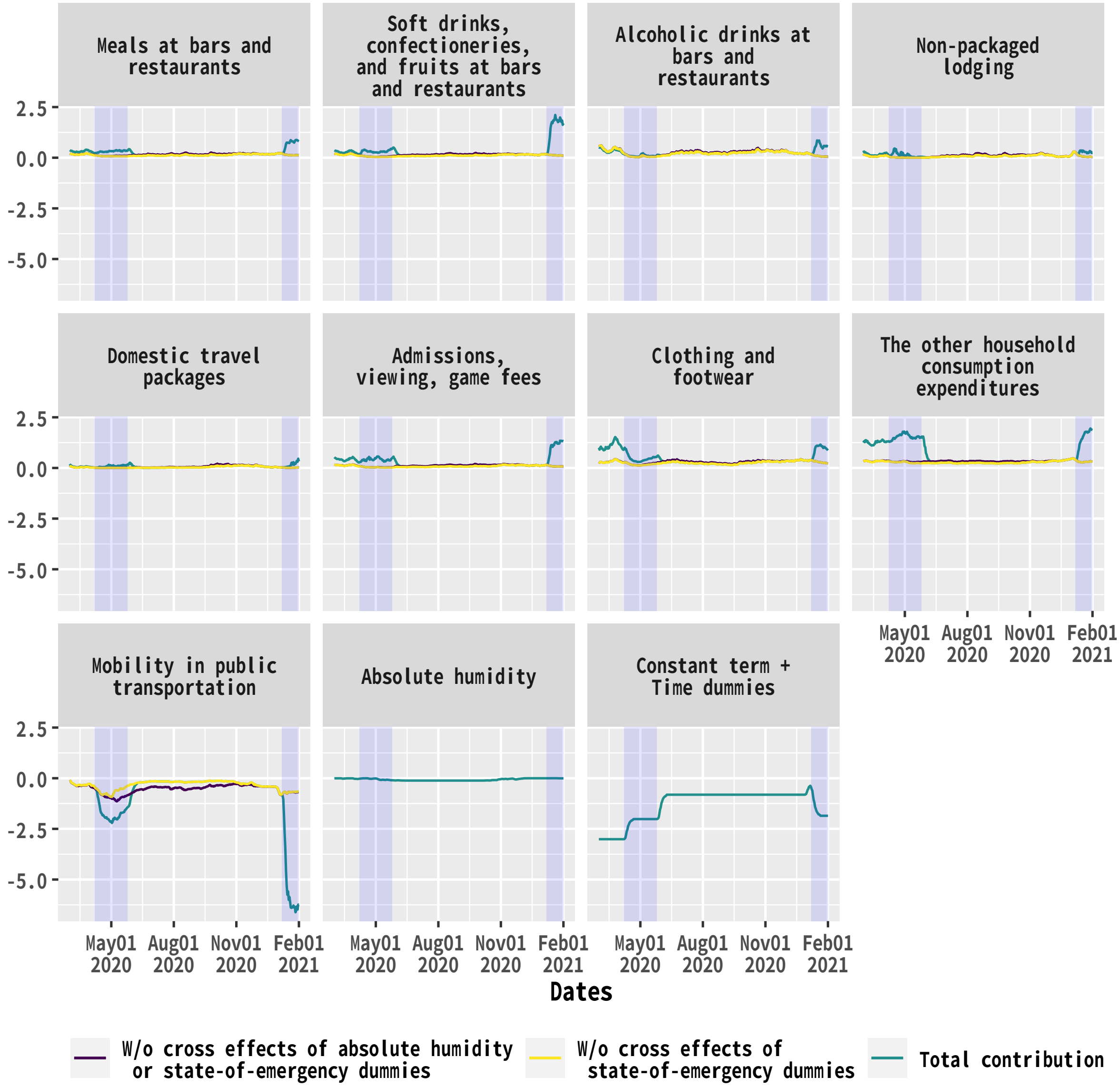

Notes: The figure shows the decomposition of fitted values of the regression when the values of time dummies related to states of emergency are unchanged from those used for the estimation of the regression model. Each panel shows the product of an explanatory variable and the posterior mean of the corresponding regression coefficient. The sample period shown in the figure is from March 1, 2020, to February 1, 2021. For household expenditures and mobility in public transportation, "W/o cross effect of absolute humidity or state-of-emergency dummies" indicates the posterior mean of $\gamma_j F(X_{j,t})$ in Eq (7), "W/o cross effect of state-of-emergency dummies" indicates the posterior mean of

$\gamma_j F(X_{j,t}) + \theta_j F(D_{AH,t} X_{j,t})$ in Eq (7), and "Total contribution" indicates the posterior mean of $\gamma_j F(X_{j,t}) + \theta_j F(D_{AH,t} X_{j,t}) + \sum_{i=1}^{3} \psi_{ij} F(D_{SoE,i,t} X_{j,t})$ in Eq (7) on each date. Each shadowed period indicates a state of emergency.
