# Supplementary material for: Associations between components of household expenditures and the rate of change in the number of new confirmed cases of COVID-19 in Japan: Time-series analysis
Source: PLoS One. 2022 Apr 14;17(4):e0266963. doi: 10.1371/journal.pone.0266963 (PMC9009719; doi:10.1371/journal.pone.0266963)
Supplement: S4 Fig — (PDF) [file pone.0266963.s011.pdf]

S4 FIG. Fitted values of the regression when time dummies related to states of emergency are set to zero.

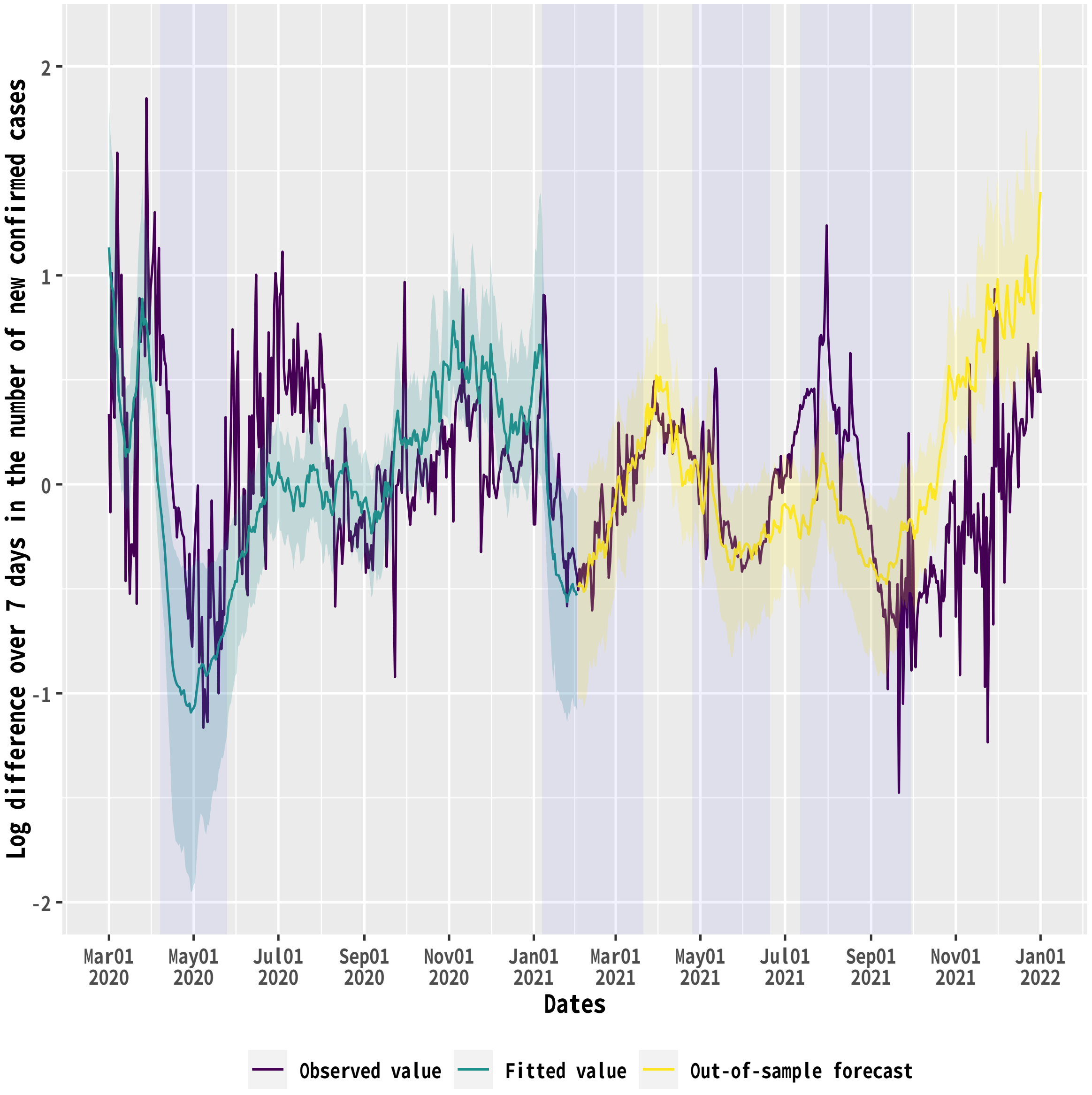

Notes: The figure shows the fitted values of the regression when time dummies for the period before the first state of emergency, the first state of emergency, and the second state of emergency are set to zero without changing the posterior means of regression coefficients. The dependent variable is the log difference over 7 days in the number of new confirmed cases of COVID-19 in Japan. The sample period shown in the figure is from March 1, 2020, to January 1, 2022. For each of the fitted values and the out-of-sample forecasts, the solid line is the posterior mean and the shadowed area indicates the 95% credible interval on each date. Each shadowed period indicates a state of emergency.
