# Supplementary material for: Associations between components of household expenditures and the rate of change in the number of new confirmed cases of COVID-19 in Japan: Time-series analysis
Source: PLoS One. 2022 Apr 14;17(4):e0266963. doi: 10.1371/journal.pone.0266963 (PMC9009719; doi:10.1371/journal.pone.0266963)
Supplement: S5 Fig — (PDF) [file pone.0266963.s012.pdf]

S5 FIG. Comparison of the "real" values of classified components of household expenditures per household in Japan over 2019-2021.

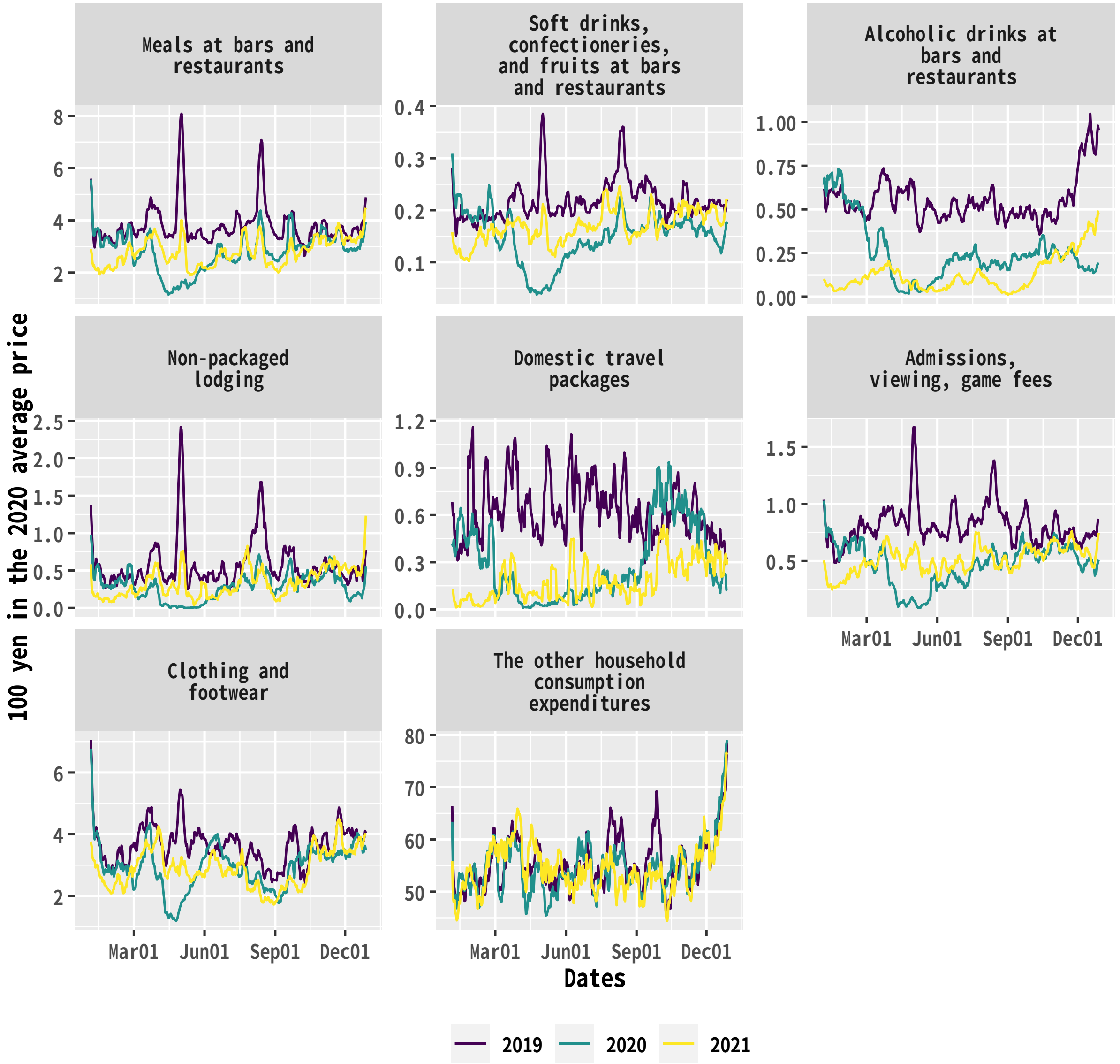

Notes: Each panel shows a component of real household expenditures per household divided by the corresponding consumer price index on each date. The unit of each series is normalized to 100 yen at the average price of each component of household expenditures in 2020. Each panel shows 7-day centered moving averages.
