# Supplementary material for: Associations between components of household expenditures and the rate of change in the number of new confirmed cases of COVID-19 in Japan: Time-series analysis
Source: PLoS One. 2022 Apr 14;17(4):e0266963. doi: 10.1371/journal.pone.0266963 (PMC9009719; doi:10.1371/journal.pone.0266963)
Supplement: Attachment — Submitted filename: Response to reviewers.docx [file pone.0266963.s013.docx]

I thank Reviewer 1 and Reviewer 2 for their valuable comments. Please find below point-by-point responses to their comments.

1 Response to Reviewer 1

- Major point 1-1) and 1-2)-i)

Following the reviewer’s comments, I have added sensitivity analysis to the revised manuscript to report the estimates of a regression without any weather variable among the explanatory variables, and regressions with indicators for ambient (outside) temperature substituting the dummy variable for absolute humidity in the explanatory variables. Please see lines 412-441 on page 14 in the revised manuscript and S4 Appendix for the results of sensitivity analysis.

In the sensitivity analysis, I report the result that the out-of-sample forecast performance of the regression model is similarly good, regardless of whether no weather variable is included in the explanatory variables, or whether the dummy variable for absolute humidity is replaced with an indicator for ambient temperature. Even though the benchmark regression with the dummy variable for absolute humidity shows a slightly better out-of-sample forecast performance than the other regressions considered in the sensitivity analysis, I conclude in the revised manuscript that, using out-of-sample forecast performance for model evaluation, the present study cannot be conclusive on the importance of weather conditions for COVID-19 incidence and transmission.

In this analysis, I do not include indicators for both ambient temperature and absolute humidity in the explanatory variables at the same time, because I do not have good information to set prior distributions of the coefficients of the two variables to avoid a multi-collinearity problem, given similar fluctuations of ambient temperature and absolute humidity. I have noted in the revised manuscript that more precise identification of the effect of weather conditions on Covid-19 incidence and transmission remains a challenge for future research. Please see the Discussion section of the revised manuscript between lines 624-629 on page 19.

- Major point 1-2)-ii)

Unfortunately, household expenditure data at daily frequency are available only at national level in the government statistics (the Family Income and Expenditure Survey [45]) that this study uses, as described in lines 609-612 on page 18. To analyze the effect of population density on COVID-19 incidence and transmission, I would need regional data to have variations of population density among samples. Following the reviewer’s comment, I have noted in the Discussion section of the revised manuscript that it is important to estimate population density on the local infectiousness of household expenditures and that this issue warrants future research. Please see lines 622-624 on page 19 for this clarification.

- Major point 2

The regression model in the present study does not include the effect of vaccinations, because it is estimated using the data for the dependent variable only up to February 1, 2021. I limit the sample period for estimation in this way in order to avoid a possible structural break due to the spread of mutant strains in 2021, as described in lines 161-176 on page 6. I use data in the subsequent sample period to compute out-of-sample forecasts of the regression model for model evaluation. Because there was no large-scale vaccination effort before February 2021 in Japan, the regression model does not include the effect of vaccinations among the explanatory variables.

Nonetheless, following the reviewer’s comment, I have added a new section (titled “Preliminary regression of out-of-sample forecast errors on the Delta-variant share of new confirmed cases and the twice-vaccinated share of the population”) after the Results section in the revised manuscript to analyze the roles of the Delta-variant share of new confirmed cases and the vaccinated share of the population in explaining the out-of-sample forecast errors of the regression model. The regression analysis described in this section implies that while the Delta-variant share of new confirmed cases and the vaccinated share of the population can explain the out-of-sample forecast errors up to mid-June 2021 and from August 2021 onward, they cannot account for a rise in the rate of change in the number of new cases from mid-June to July 2021. A caveat is that this conclusion is tentative, as the regression analysis in the new section is yet to be validated by out-of-sample forecast performance. Please see lines 465-502 on pages 15-16 for the regression analysis.

- Major point 3

Following the reviewer’s comment, I have included the Delta-variant share of new confirmed cases in Japan among the explanatory variables in the regression analysis described in the response to Major point 2 in this letter. Please see lines 465-502 on pages 15-16 for the regression analysis.

- Minor point 1

Following the reviewer’s suggestion, I cite the clinical guide published by the MHLW of the Japanese government [41] instead of the CDC recommendation regarding the range of incubation periods of COVID-19. Please see lines 153-155 on pages 5-6.

Except the citation of the CDC guideline in this part, there is no other citation of foreign public health agencies outside Japan in the revised manuscript.

- Minor point 2

To improve the resolution of figures to the satisfactory level for the PLOS ONE journal, I use the PACE website (<https://pacev2.apexcovantage.com/>) to convert figures into image files, following the instruction on figures by the journal.

2 Response to Reviewer 2

- Comment 1

Yes. The typo is corrected in the revised manuscript. I thank the reviewer for pointing out the error.

- Comment 2-a

Following the reviewer’s comment, I have added a clarification in the revised manuscript that, because household expenditures can be made both online and offline, the coefficient of each explanatory variable in the regression captures the average effect of the variable. Please see lines 106-111 on page 4 for the clarification.

- Comment 2-b

Following the reviewer’s comment, I have added sensitivity analysis to the revised manuscript to report the estimation result of an alternative regression in which household expenditures for clothing and footwear in the explanatory variables include only offline purchases. I do not separate online and offline purchases for the other types of household expenditures in the explanatory variables, because they are offline services except “the other household expenditures”, even if the payments are made online, while “the other household expenditures” is the residual household expenditures other than classified household expenditures included in the explanatory variables. Please see lines 442-464 on pages 14-15 and S5 appendix for the sensitivity analysis.

In the sensitivity analysis, I report that the estimation results of the alternative regression with offline household expenditures for clothing and footwear are similar to those of the benchmark regression with total (i.e., the sum of online and offline) household expenditures for clothing and footwear. For a possible reason for this result, I refer to the fact that the online share of household expenditure for clothing and footwear had been stable during the sample period after a permanent increase in early 2020. I also mention the limitation of this interpretation at the end of the sensitivity analysis.

- Comment 3-a

A regression model with a structural break has a strength in the precise identification of the date of the structural break. A challenge for this approach is that the estimation of coefficients both before and after the structural break will be biased unless the regression model is correctly specified for the entire sample period before and after the structural break. This issue is especially challenging for the present study, because it is yet difficult to specify correctly in a regression model the effects of multiple mutant strains, some of which appeared only recently, and vaccinations, whose protective effects are decaying over time and may be different for different strains. One way to mitigate this challenge is to focus on an early part of the sample period clearly before observed structural breaks, such as the spread of mutant strains and the progress of vaccinations in 2021 in Japan. The present study takes this approach. Another benefit of this approach is that the regression model can be validated by out-of-sample forecasts (i.e., testing data), because not all the available sample period is used for the estimation of the regression model. In contrast, model validation by out-of-sample forecasts is usually difficult for a regression model with a structural break, because a structural break is typically introduced into a regression model to use all the available sample period for in-sample fitting.

Thus, the present study has some benefits that a regression model with a structural break does not have, and vice versa. Following the reviewer’s comment, I mention the benefit of estimating a regression model with a structural break as an issue for future research in the Discussion section in the revised manuscript. Please see lines 631-642 on page 19 for this clarification.

- Comment 3-b

Following the reviewer’s comment, I have added a new section (titled “Preliminary regression of out-of-sample forecast errors on the Delta-variant share of new confirmed cases and the twice-vaccinated share of the population”) after the Results section in the revised manuscript to analyze the roles of the Delta-variant share of new confirmed cases and the vaccinated share of the population in explaining the out-of-sample forecast errors of the regression model. Please see lines 465-502 on pages 15-16 for this analysis.

- Comment 3-c

If regression coefficients had changed significantly between 2020 and 2021, then the out-of-sample forecasts of the regression would not fit well the realized values of the dependent variable in the out-of-sample forecast period from February 2, 2021, onward. Nonetheless, it is possible that a temporary change in regression coefficients took place during the summer of 2020, for which the in-sample fit of the regression is low. Following the reviewer’s comment, this issue is added as a limitation of the present study in the Discussion section. Please see lines 643-656 on page 19.

- Comment 4

As stated in the reviewer’s comment, it is possible that people changed their behavior during each state of emergency. To capture the effect of such a behavioral change on the infectiousness of household expenditures, a time dummy for each state of emergency during the sample period is introduced in the regression model. Please see lines 119-122 on page 4 for this clarification. Because a time dummy only captures a time-varying average, the estimated coefficient of the time dummy for each state of emergency cannot tell the reason for the estimated time effect. Also, as will be described in the response to the reviewer’s comment 8, the estimated coefficients of time dummies for the states of emergencies are likely to suffer overfitting. Given this result, I do not try to interpret the estimated values of these coefficients in the revised manuscript.

- Comment 5

Because of lack of daily data for single-person households, it cannot be known if expenditures by single-person households were just proportional to expenditures by households with two or more persons, or if including them would improve the fit of the regression model to data significantly, such as the low in-sample fit during the summer of 2020. The present study reports that the regression model with expenditures by households with two or more persons can be validated by a good fit of out-of-sample forecasts of the regression. Following the reviewer’s comment, I mention the possibility of improving the fit of the regression model by including single-person household data for an explanatory variable in the Discussion section of the revised manuscript. Please see lines 651-656 on page 19 for this clarification.

- Comment 6

The presence of a lag in the sample correlation between the log difference over 7 days in the number of new confirmed cases and each large category of household expenditures shown in Table 1 is partly due to the presence of incubation periods after infections. Because it is a sample correlation, differences in lag lengths across different categories of household expenditures can be also due to differences in unobserved activities associated with each category of household expenditures. The possible effects of indirectly associated consumer activities for each classified component of household expenditures in the explanatory variables are mentioned in the Discussion section of the revised manuscript. Please see lines 571-575 on page 18 for this description.

In addition, trying to remove the effect of such indirect channels and extract the direct effect of each household expenditure on incidence of an infection, the regression model in the present study constrains that household expenditures in the explanatory variables can affect the dependent variable on a future date only in proportion to the exogenous distribution of incubation periods. This use of the distribution of incubation periods in the regression model is described in lines 78-86 on page 3.

- Comment 7-a

Regarding the use of the word “anomaly” in the previous manuscript, I intended to simply mean that the regression in the manuscript fails to account for the second wave of COVID-19 infection in Japan, rather than that the regression model is perfect. Following the reviewer’s comment, the sentence referred to by this comment is removed from the revised manuscript.

- Comment 7-b

Various possibilities of improving the fit of the regression model to data by incorporating a larger set of explanatory variables are mentioned in the Discussion section in the revised manuscript. Please see responses to the reviewer’s earlier comments, and also lines 643-656 on page 19. Also, I have added a new section on the regression of the out-of-sample forecast errors on the Delta-variant share of new confirmed cases and the vaccinated share of the population, as described in the response to the reviewer’s comment 3-b.

- Comment 8

Overfitting of a regression occurs when regression coefficients estimated by the in-sample fit of the regression produce imprecise out-of-sample forecasts. Following the reviewer’s comment, I have added S2 Fig (a figure in an online appendix) to the revised manuscript to report the out-of-sample forecasts of the regression in which a time dummy for the second state of emergency is not set to zero in the out-of-sample forecast period. This figure demonstrates a poor fit of the out-of-sample forecasts of the regression, despite a good in-sample fit of the regression. Please see lines 686-687 on page 20 for confirmation.

- Comment 9-a

Following the reviewer’s comment, the subsection of the Discussion section referred to by the reviewer is moved to an earlier section, which describes the regression of out-of-sample forecast errors on the Delta-variant share of new confirmed cases and the vaccinated share of the population. Please see lines 465-502 on pages 15-16 for confirmation.

- Comment 9-b

Following the reviewer’s comment, all the figures in the Discussion section in the previous manuscript is either moved to earlier sections or online appendices. In the revised manuscript, there is no figure included in the main text of the Discussion section. Please see lines 503-656 on pages 16-19 for confirmation.

- Comment 10

Following the reviewer’s comment, I have added “except a low in-sample fit of the regression for the summer of 2020” after the expression in the conclusion criticized by the reviewer. I have also fully revised the conclusion part of the abstract to avoid using the phrase “stable association”. Please see lines 661-662 on page 19 and the abstract on page 1 for confirmation.
