# Supplementary material for: Associations between components of household expenditures and the rate of change in the number of new confirmed cases of COVID-19 in Japan: Time-series analysis
Source: PLoS One. 2022 Apr 14;17(4):e0266963. doi: 10.1371/journal.pone.0266963 (PMC9009719; doi:10.1371/journal.pone.0266963)
Supplement: Attachment — Submitted filename: Response to reviewers in the second revision.docx [file pone.0266963.s014.docx]

I thank Reviewer 1 and Reviewer 2 for their suggestions to improve the manuscript. Please find below point-by-point responses to their comments.

1 Response to Reviewer 1

Following the reviewer’s suggestion, the Conclusion section of the abstract is changed to a more substantial explanation of this study’s contribution: “The estimated model can be potentially useful in simulating changes in the number of new confirmed cases due to household spending on retail services, if it can be adjusted to real-time developments of mutant strains and vaccinations. Such simulations would help in designing cost-efficient government interventions.”

To accommodate the longer conclusion section within the word limit for the abstract, the Objective section of the previous abstract is dropped from the revised manuscript. Because the Objective section overlapped the Methods section in the previous abstract, there is no significant loss of information caused by this revision.

2 Response to Reviewer 2
1) Following the reviewer’s suggestion, the title of the subsection is changed from “Limitations” to “Limitations and strengths” in the revised manuscript. Please see Line 552 on page 17 for confirmation.

Furthermore, the Discussion and Conclusion section has been edited by a professional English editor at Editage, an academic editing company. The editor is a native English speaker according to the company. Most of the English editing is incorporated in the revised manuscript, except a few jargons such as “Delta variant” and “new confirmed cases”, and a few expressions that are kept to be consistent with the earlier sections of the revised manuscript. I believe the Discussion and Conclusion section has become easier to read, as the reviewer suggested.

2) Following the reviewer’s suggestion to make the Conclusion section more substantial, the end of the Conclusion section is revised to clarify that the findings reported in this study can be potentially used for designing cost-efficient government interventions in consumer activities to contain the spread of COVID-19, and also for predicting seasonal fluctuations in the number of new confirmed cases of COVID-19. Please see lines 674-681 on page 20 for confirmation. For the same purpose, the Conclusion section of the abstract is also revised, as described in the response to Reviewer 1 above.
